# Supplementary figures and images for: KDM6B interacts with TFDP1 to activate P53 signaling in regulating mouse palatogenesis
Source: eLife. 2022 Feb 25;11:e74595. doi: 10.7554/eLife.74595 (PMC9007587; doi:10.7554/eLife.74595)

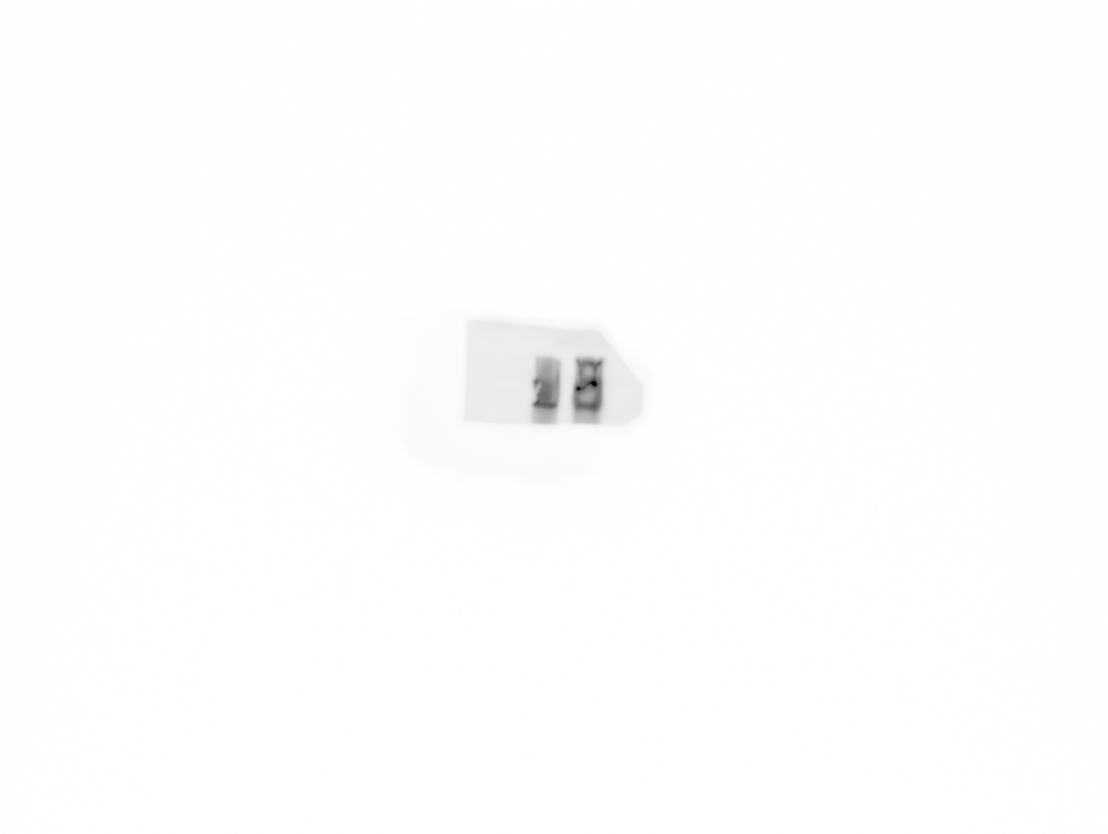

Supplement: Figure 1—figure supplement 1—source data 1. [file elife-74595-fig1-figsupp1-data1.zip › Figure 1-figure supplement 1-source data 1 for 1M/Figure 1-figure supplement 1-source data 1 for 1M.tif]

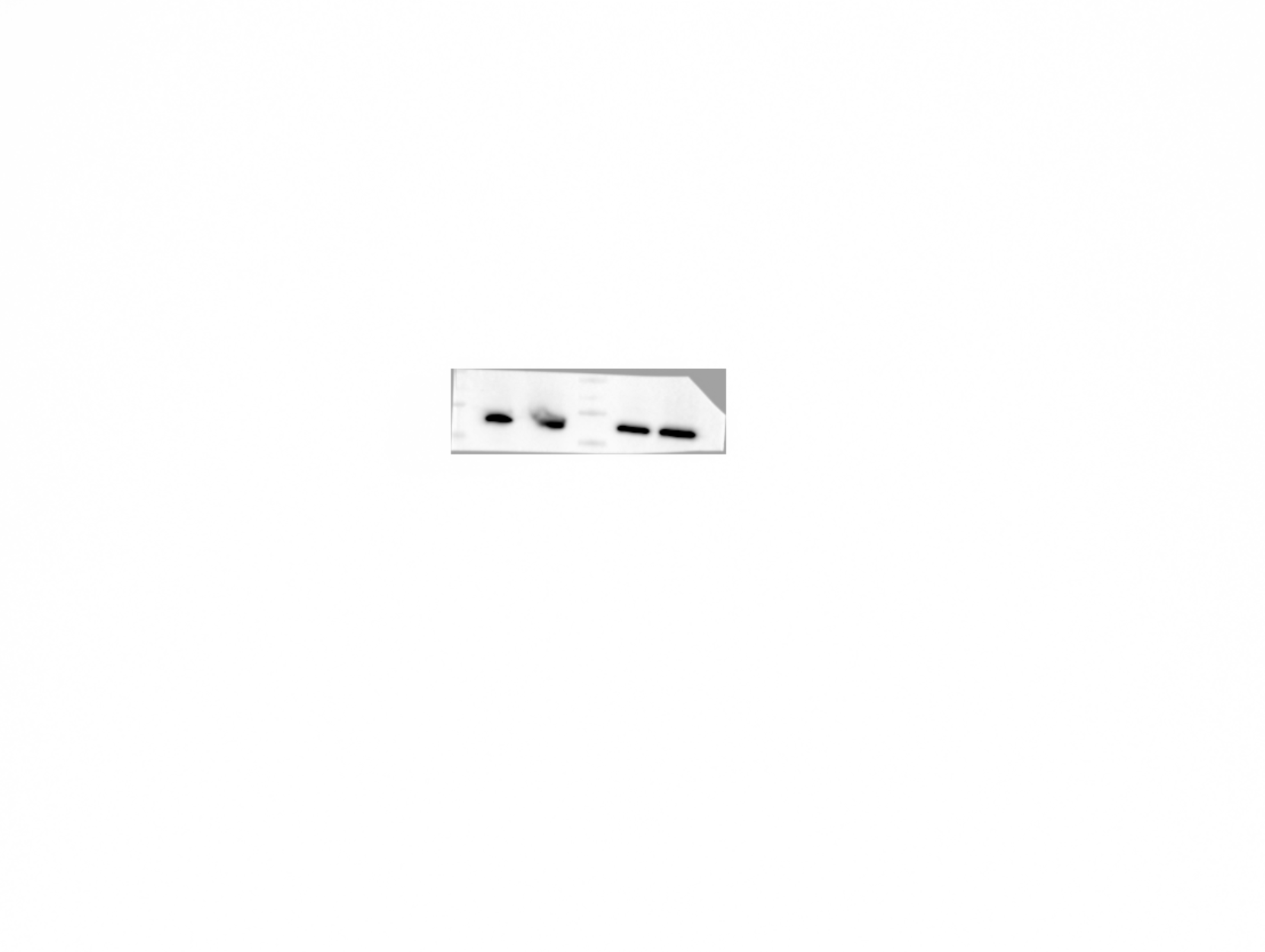

Supplement: Figure 1—figure supplement 1—source data 1. [file elife-74595-fig1-figsupp1-data1.zip › Figure 1-figure supplement 1-source data 1 for 1M/Figure 1-figure supplement 1-source data 2 for 1M.tif]

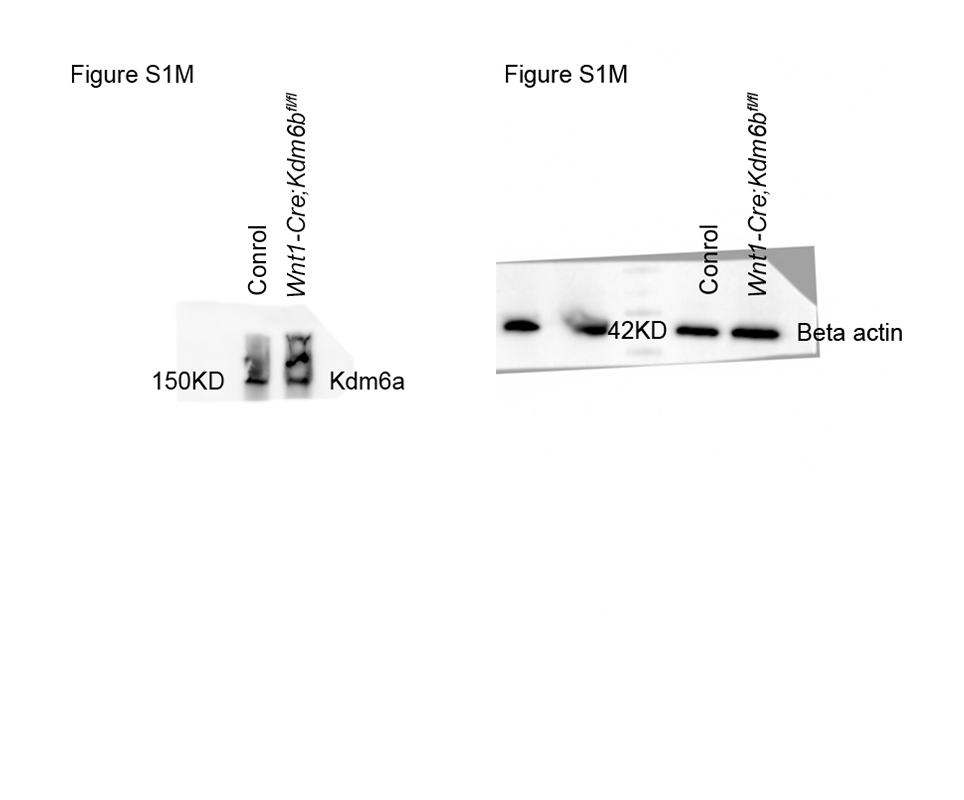

Supplement: Figure 1—figure supplement 1—source data 1. [file elife-74595-fig1-figsupp1-data1.zip › Figure 1-figure supplement 1-source data 1 for 1M/Figure 1-figure supplement 1-source data 3 for 1M.tif]

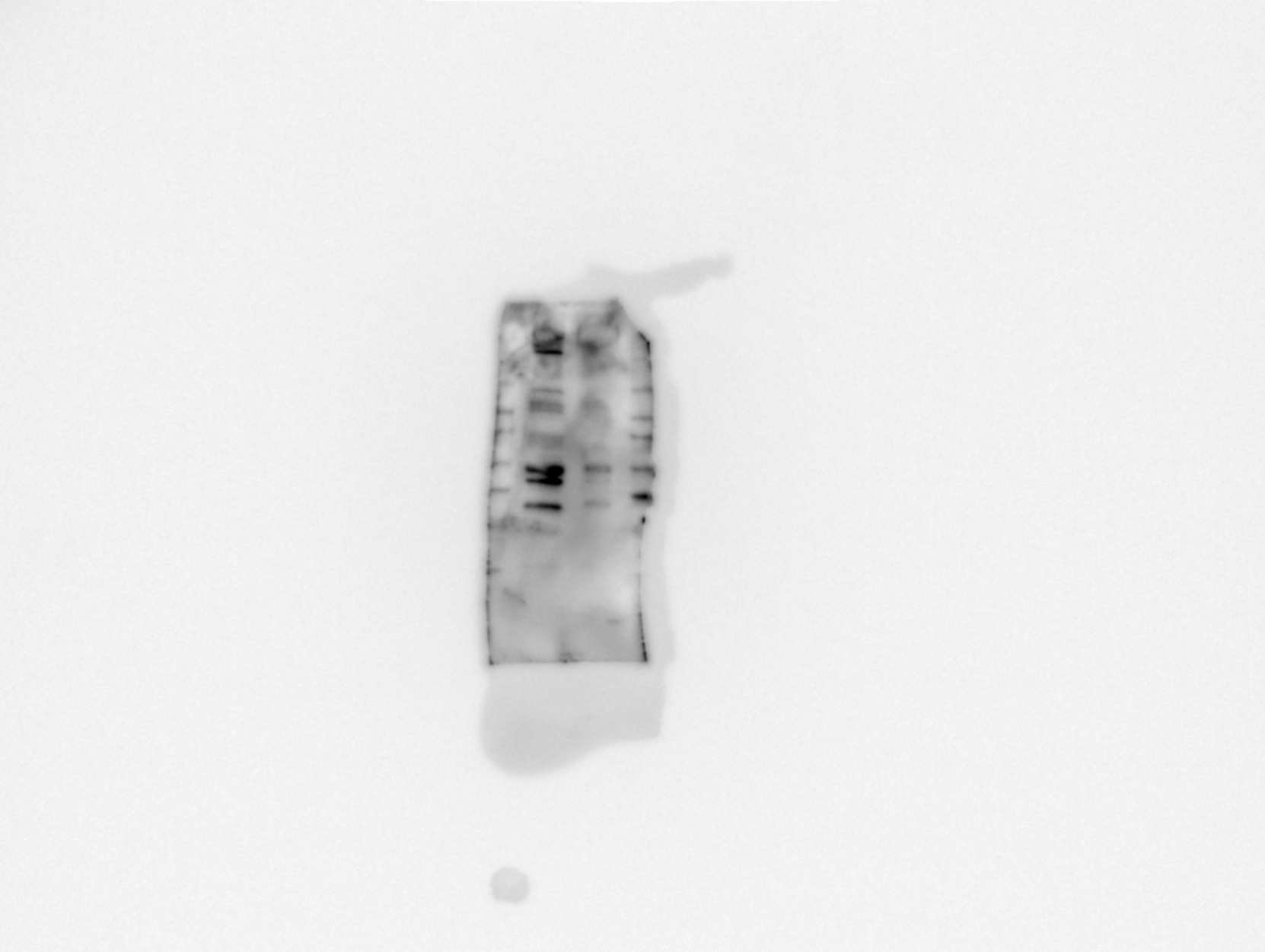

Supplement: Figure 1—figure supplement 1—source data 2. [file elife-74595-fig1-figsupp1-data2.zip › Figure 1-figure supplement 1-source data 2 for 1N/Figure 1-figure supplement 1-source data 4 for 1N.tif]

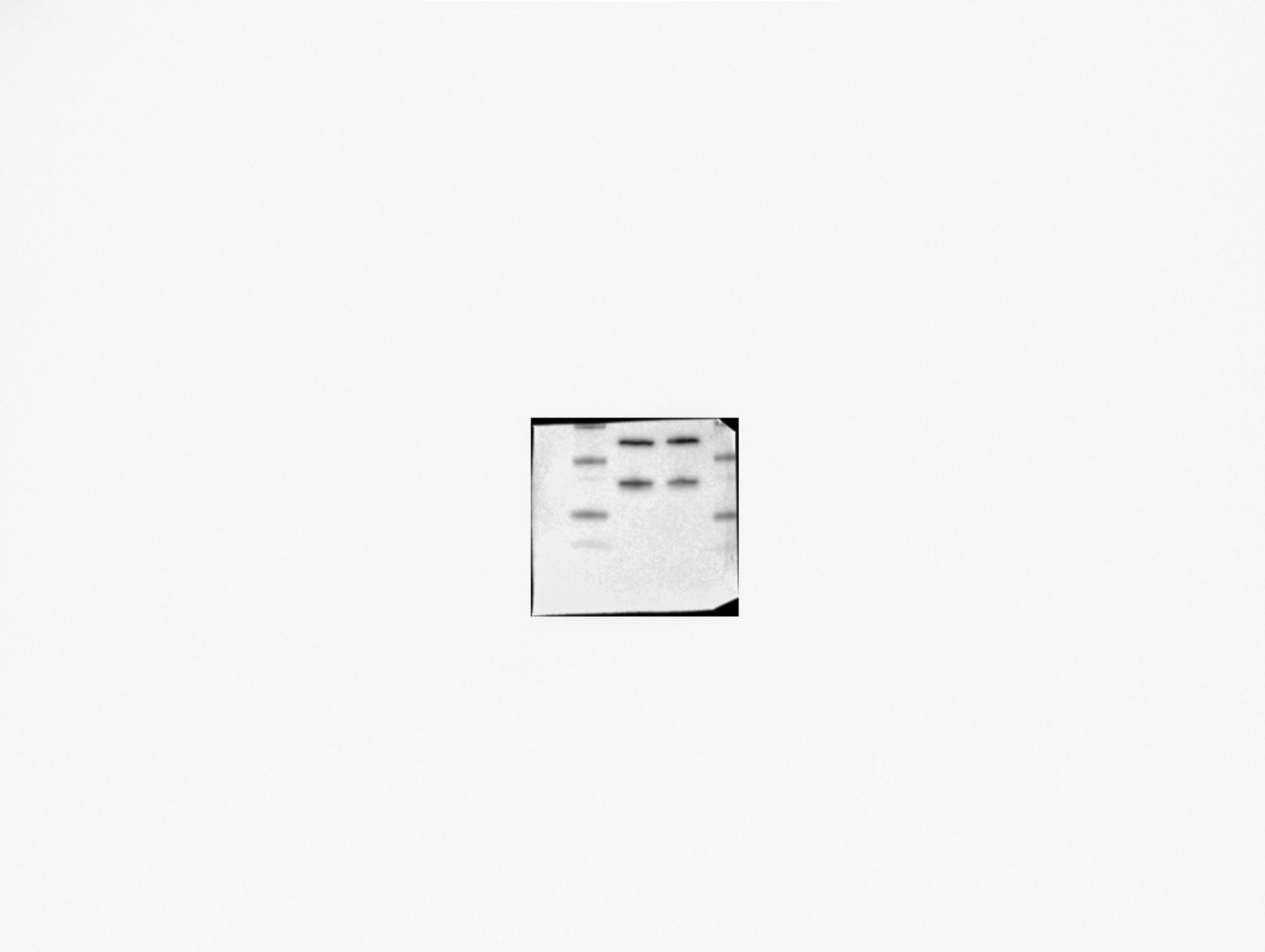

Supplement: Figure 1—figure supplement 1—source data 2. [file elife-74595-fig1-figsupp1-data2.zip › Figure 1-figure supplement 1-source data 2 for 1N/Figure 1-figure supplement 1-source data 5 for 1N.tif]

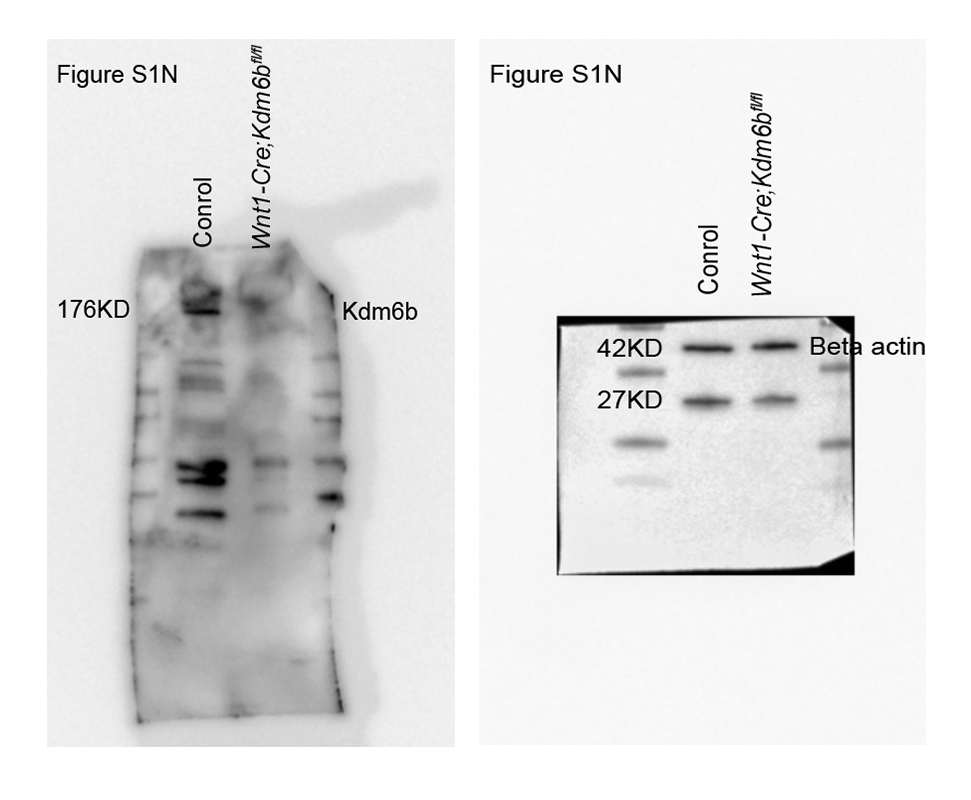

Supplement: Figure 1—figure supplement 1—source data 2. [file elife-74595-fig1-figsupp1-data2.zip › Figure 1-figure supplement 1-source data 2 for 1N/Figure 1-figure supplement 1-source data 6 for 1N.tif]

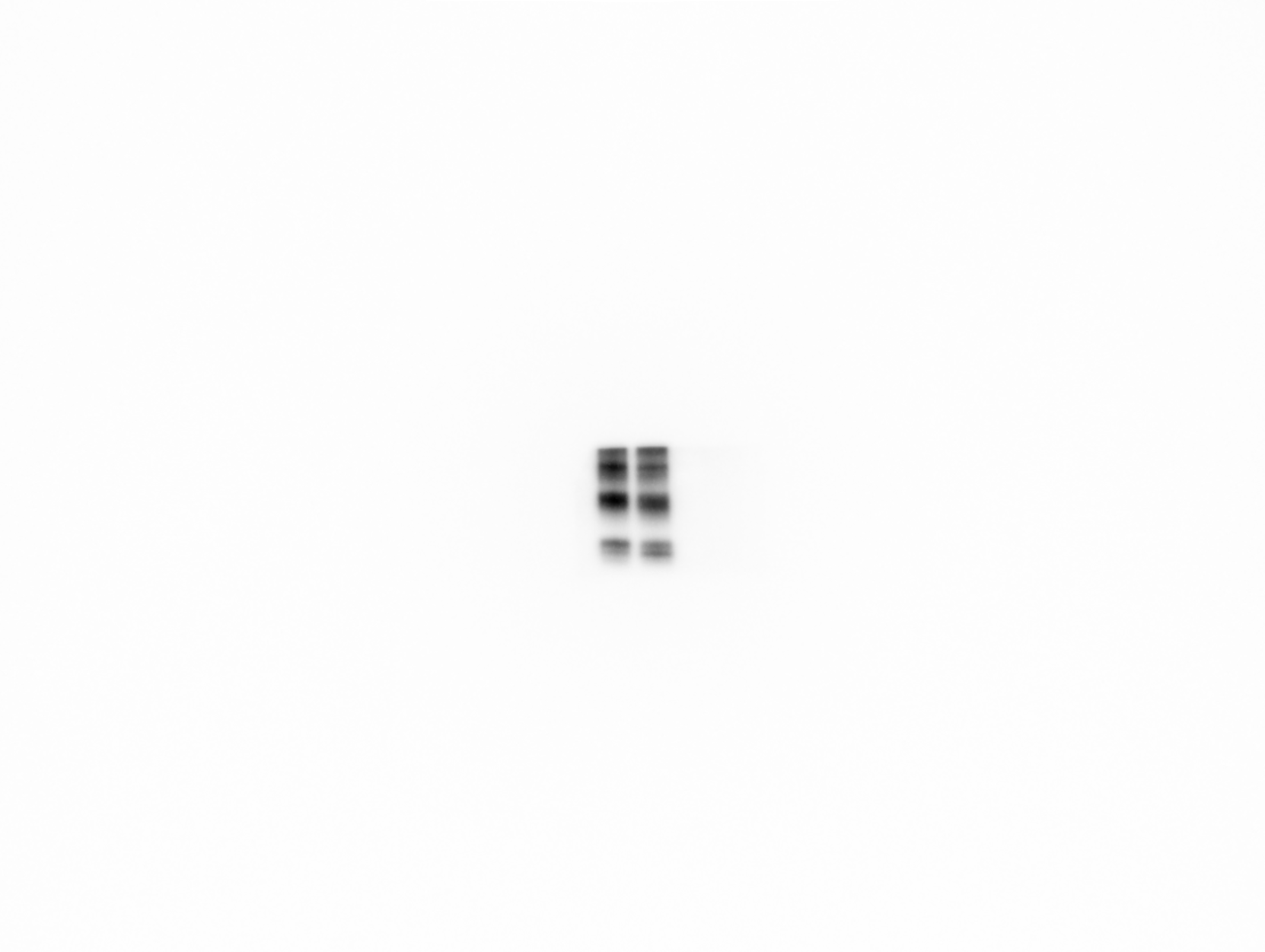

Supplement: Figure 4—figure supplement 1—source data 4. [file elife-74595-fig4-figsupp1-data4.zip › Figure 4-figure supplement 1-source data 4 for 1F/Figure 4-figure supplement 1-source data 4 for 1F.tif]

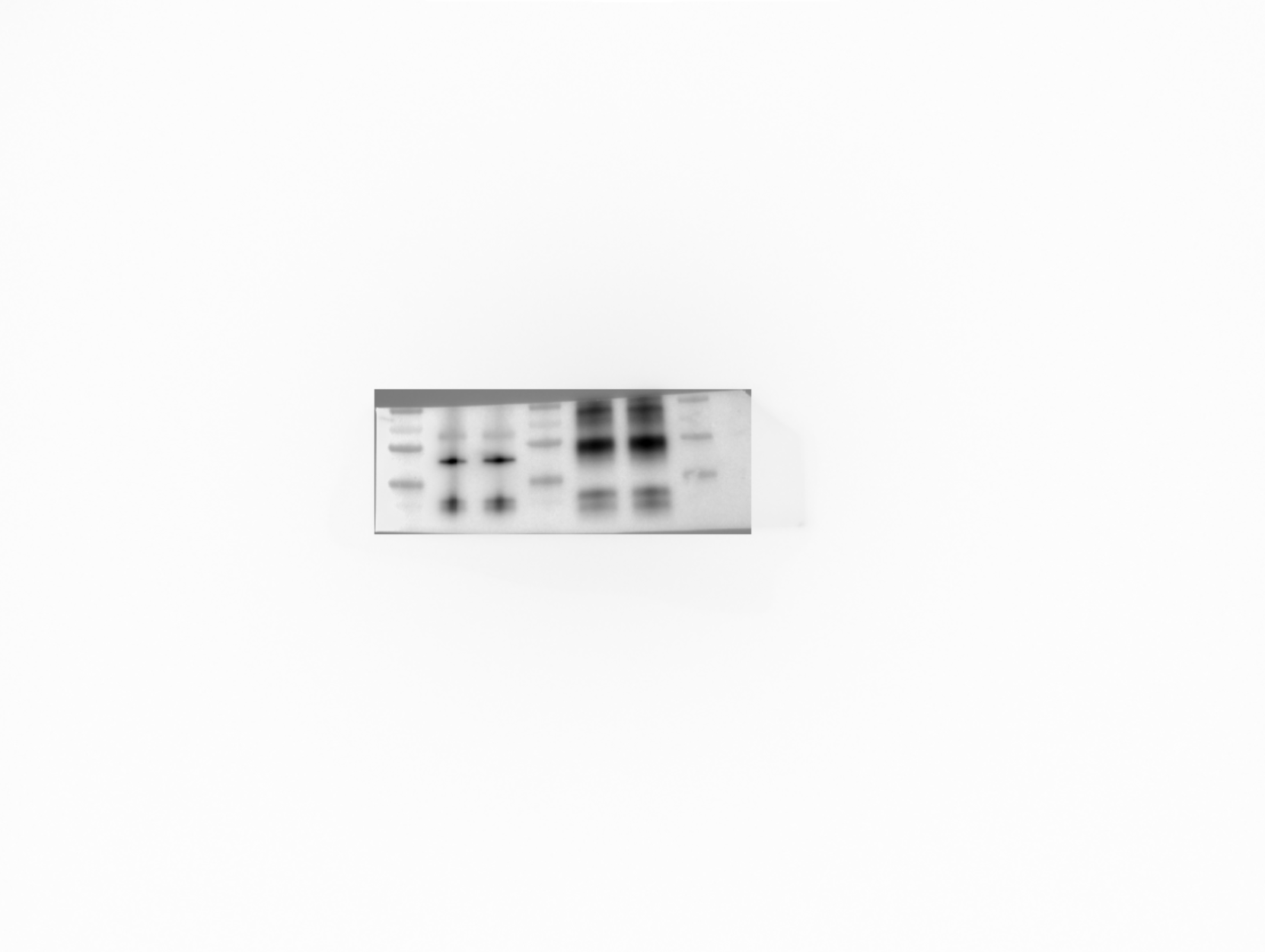

Supplement: Figure 4—figure supplement 1—source data 4. [file elife-74595-fig4-figsupp1-data4.zip › Figure 4-figure supplement 1-source data 4 for 1F/Figure 4-figure supplement 1-source data 5 for 1F.tif]

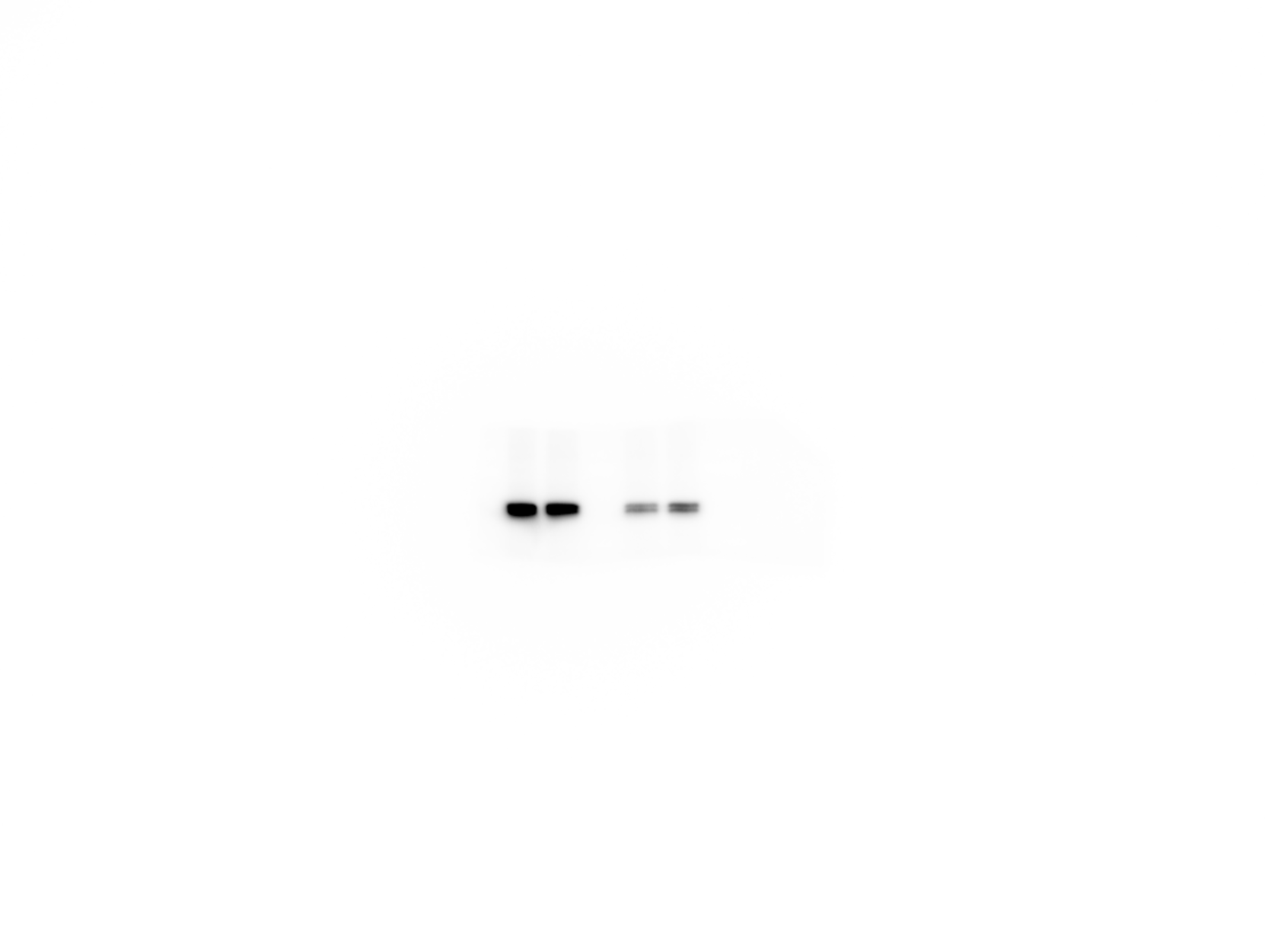

Supplement: Figure 4—figure supplement 1—source data 4. [file elife-74595-fig4-figsupp1-data4.zip › Figure 4-figure supplement 1-source data 4 for 1F/Figure 4-figure supplement 1-source data 6 for 1F.tif]

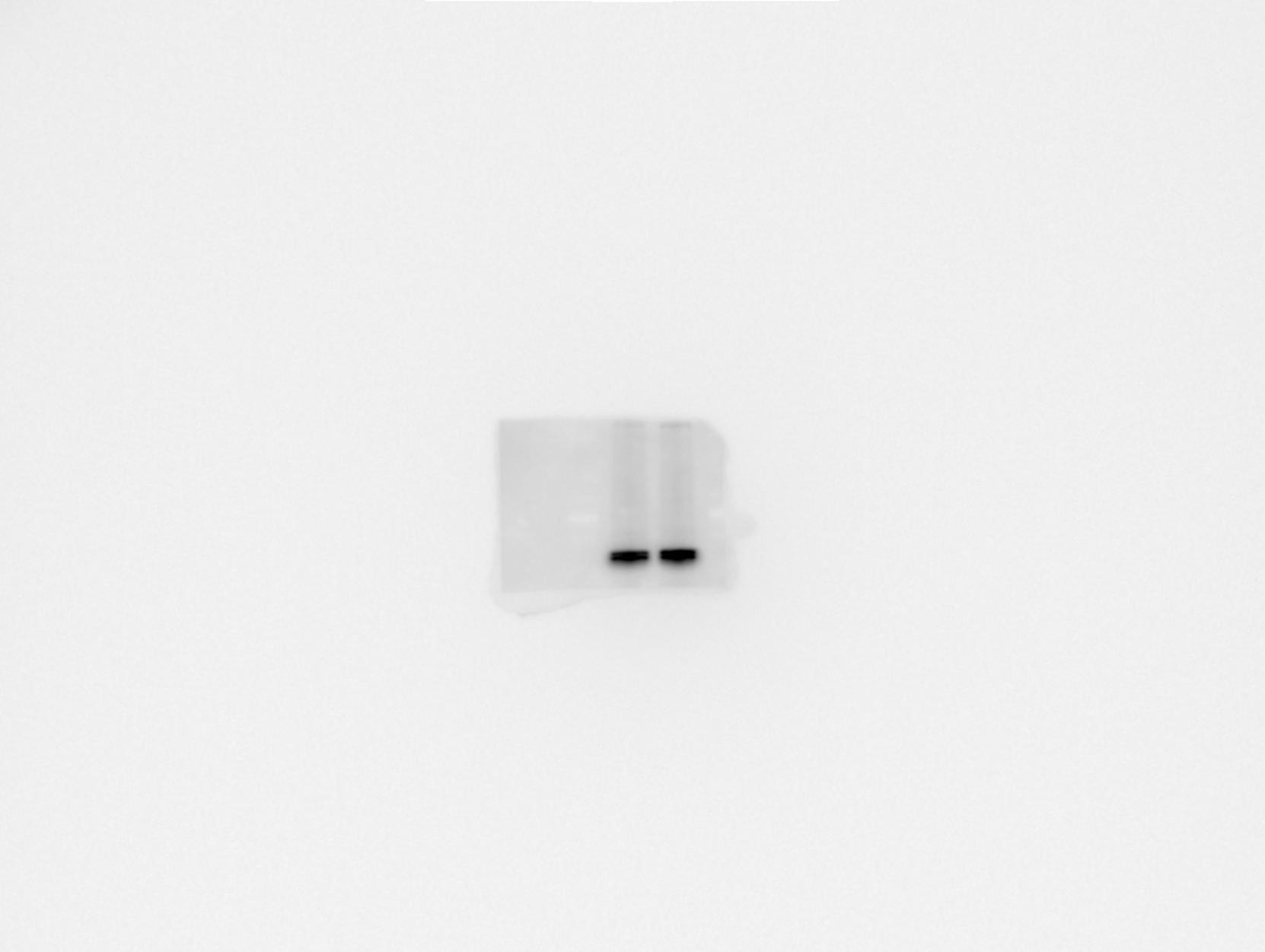

Supplement: Figure 4—figure supplement 1—source data 4. [file elife-74595-fig4-figsupp1-data4.zip › Figure 4-figure supplement 1-source data 4 for 1F/Figure 4-figure supplement 1-source data 7 for 1F.tif]

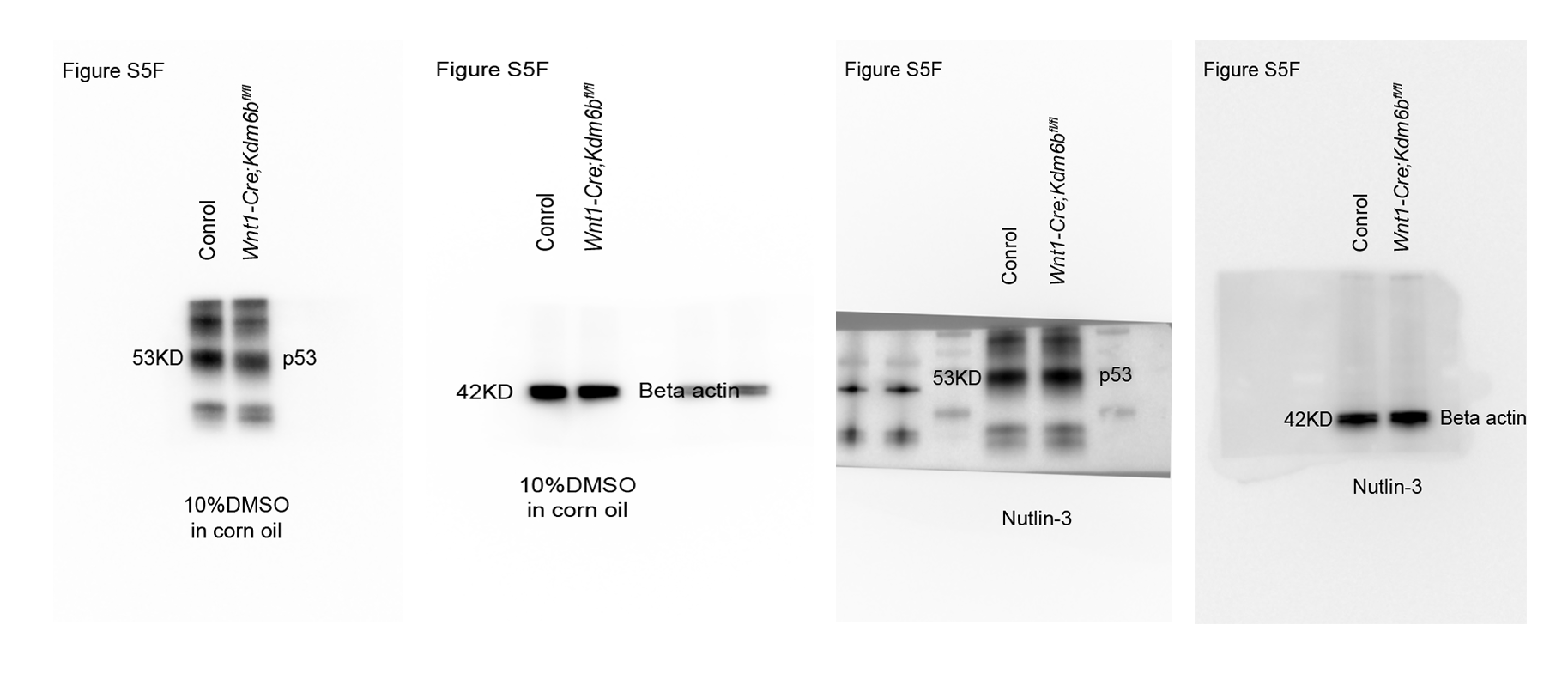

Supplement: Figure 4—figure supplement 1—source data 4. [file elife-74595-fig4-figsupp1-data4.zip › Figure 4-figure supplement 1-source data 4 for 1F/Figure 4-figure supplement 1-source data 8 for 1F.tif]

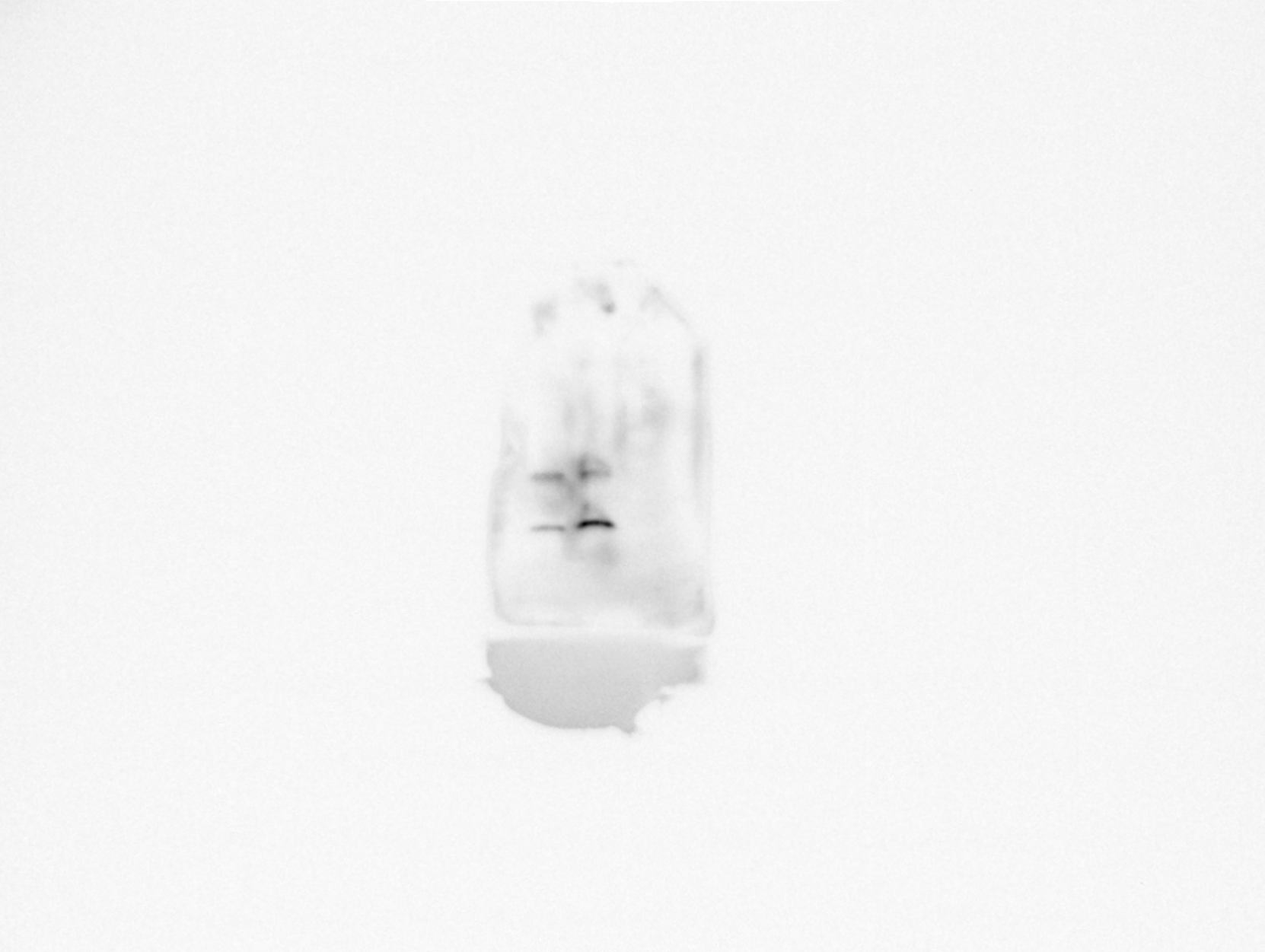

Supplement: Figure 5—source data 1. [file elife-74595-fig5-data1.zip › Figure 5-source data 1 for 5E/Figure 5-source data 1 for Figure 5E.tif]

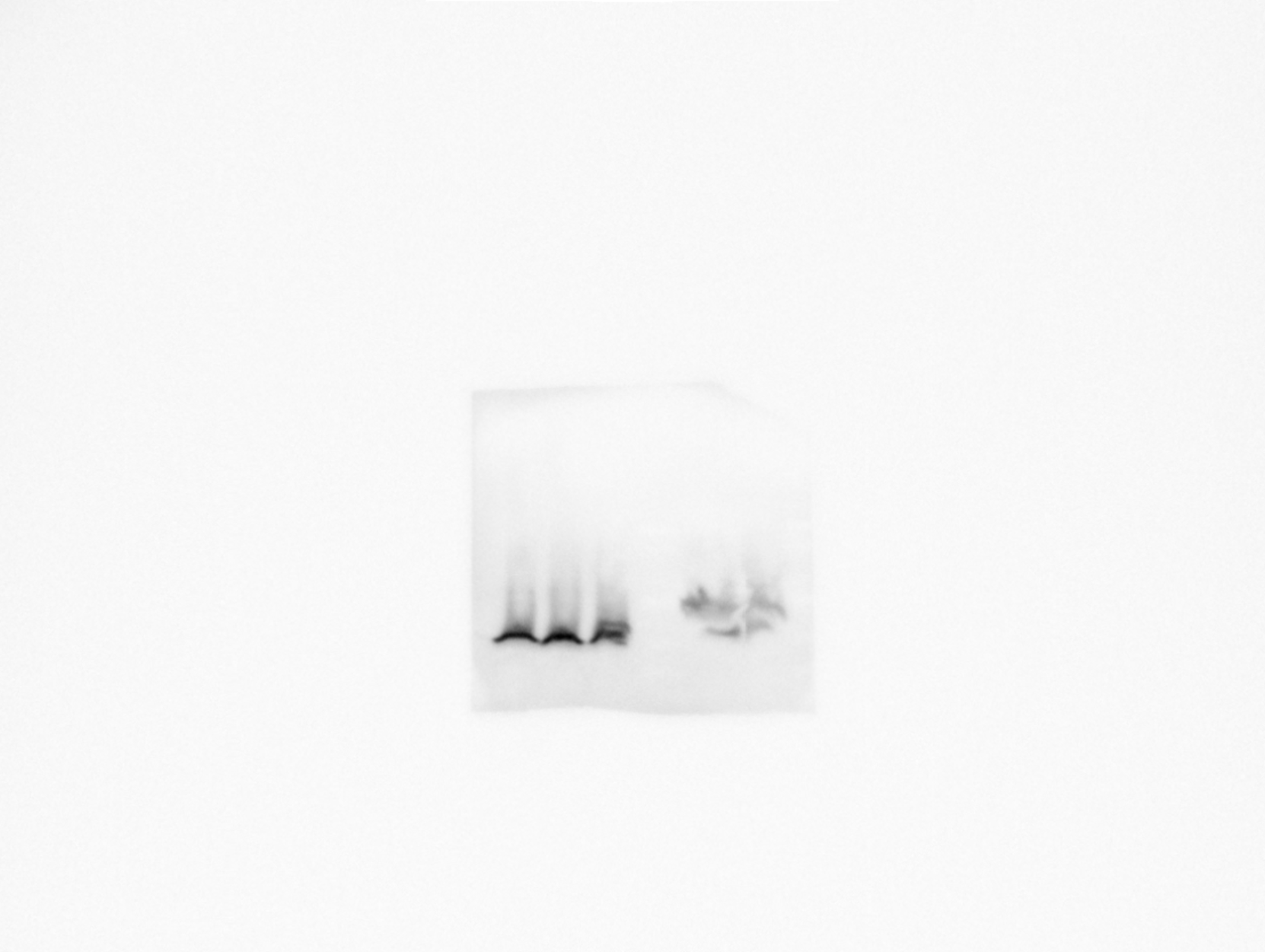

Supplement: Figure 5—source data 1. [file elife-74595-fig5-data1.zip › Figure 5-source data 1 for 5E/Figure 5-source data 2 for Figure 5E.tif]

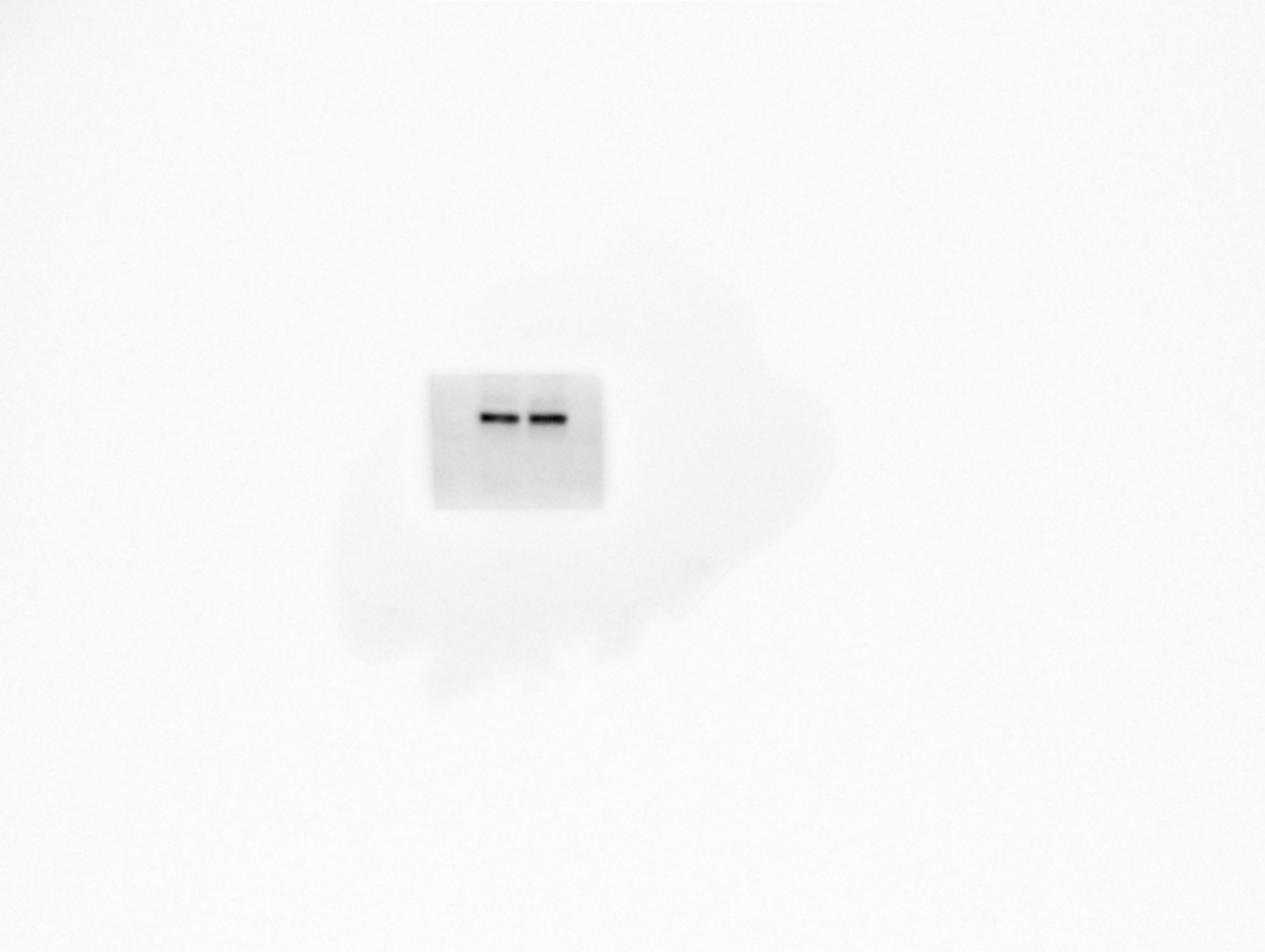

Supplement: Figure 5—source data 1. [file elife-74595-fig5-data1.zip › Figure 5-source data 1 for 5E/Figure 5-source data 3 for Figure 5E.tif]

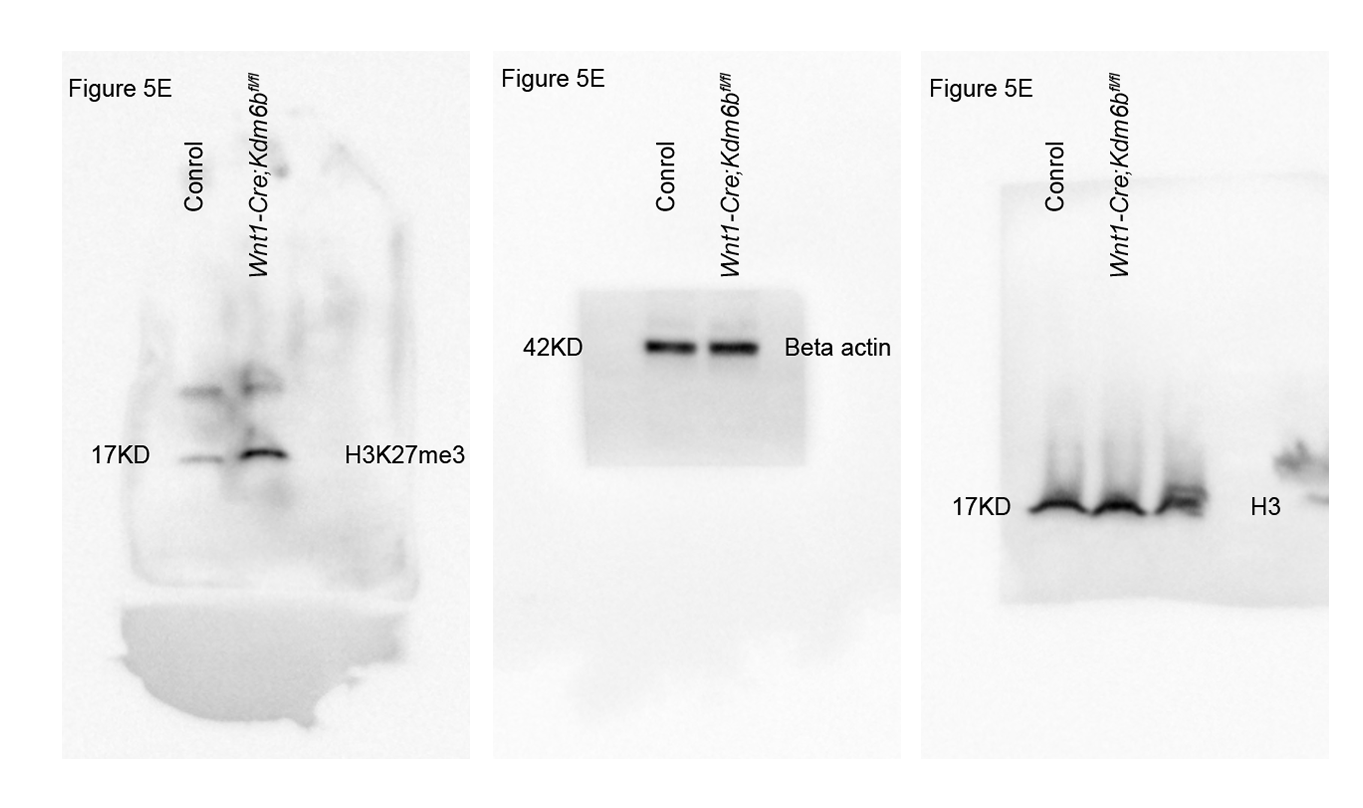

Supplement: Figure 5—source data 1. [file elife-74595-fig5-data1.zip › Figure 5-source data 1 for 5E/Figure 5-source data 4 for Figure 5E.tif]

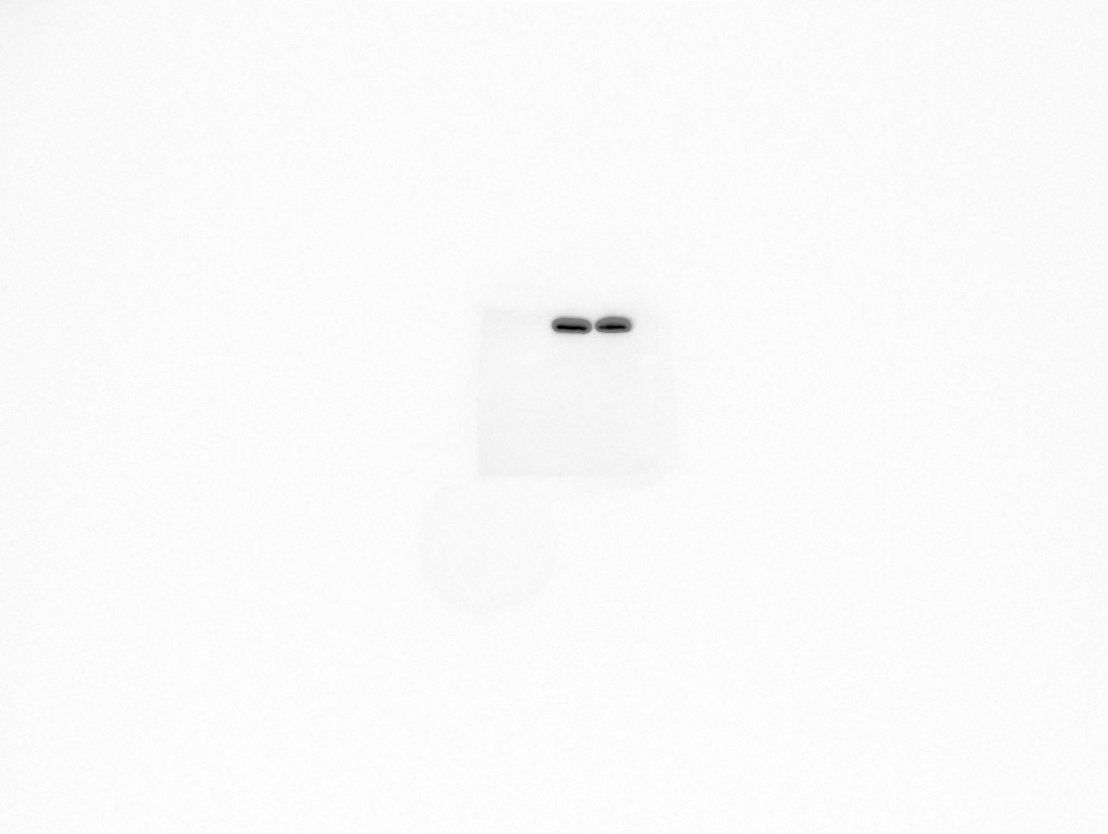

Supplement: Figure 5—source data 2. [file elife-74595-fig5-data2.zip › Figure 5-source data 2 for 5J/Figure 5-source data 5 for Figure 5J.tif]

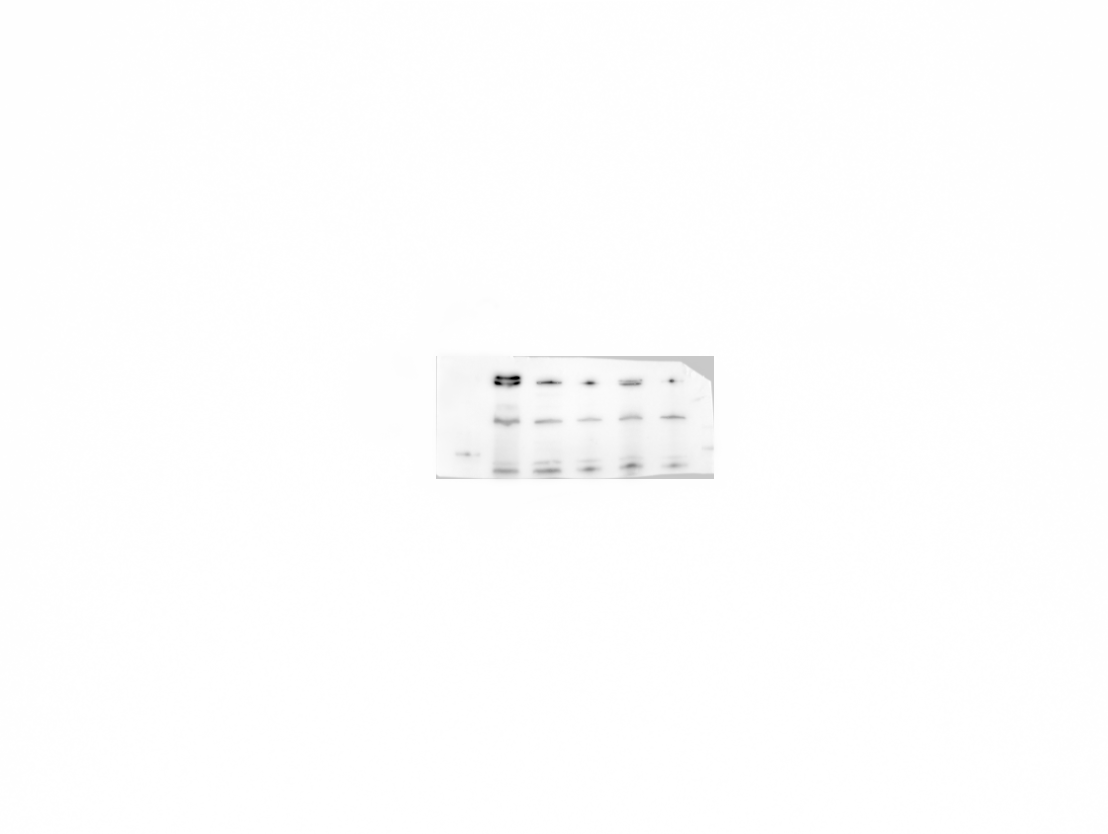

Supplement: Figure 5—source data 2. [file elife-74595-fig5-data2.zip › Figure 5-source data 2 for 5J/Figure 5-source data 6 for Figure 5J.tif]

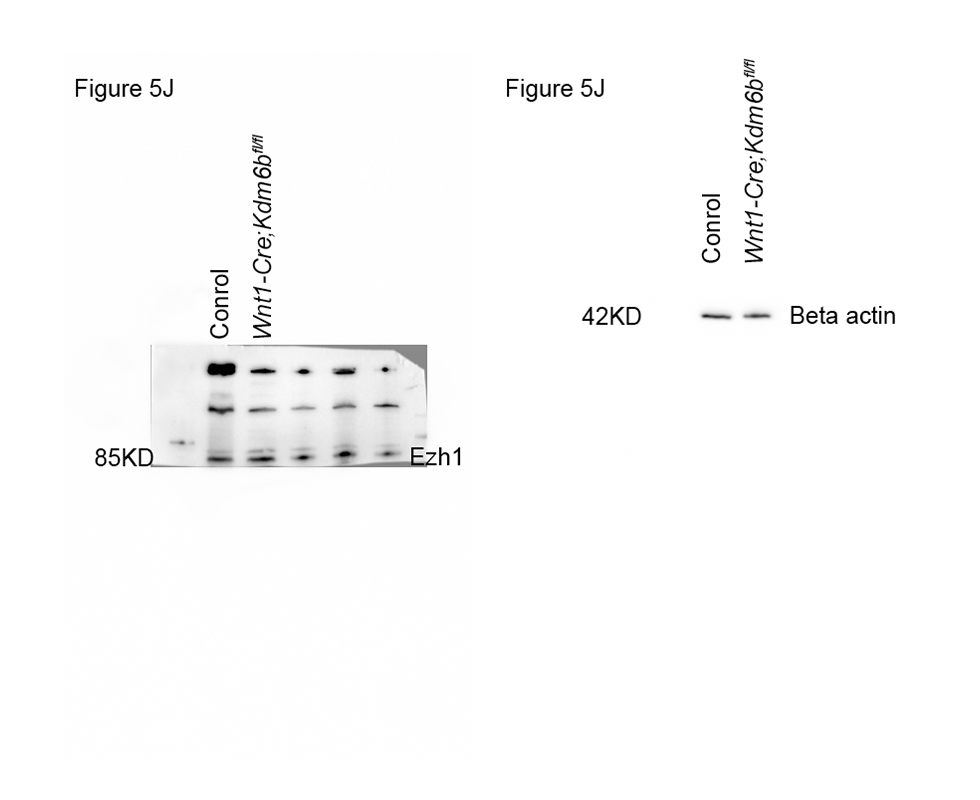

Supplement: Figure 5—source data 2. [file elife-74595-fig5-data2.zip › Figure 5-source data 2 for 5J/Figure 5-source data 7 for Figure 5J.tif]

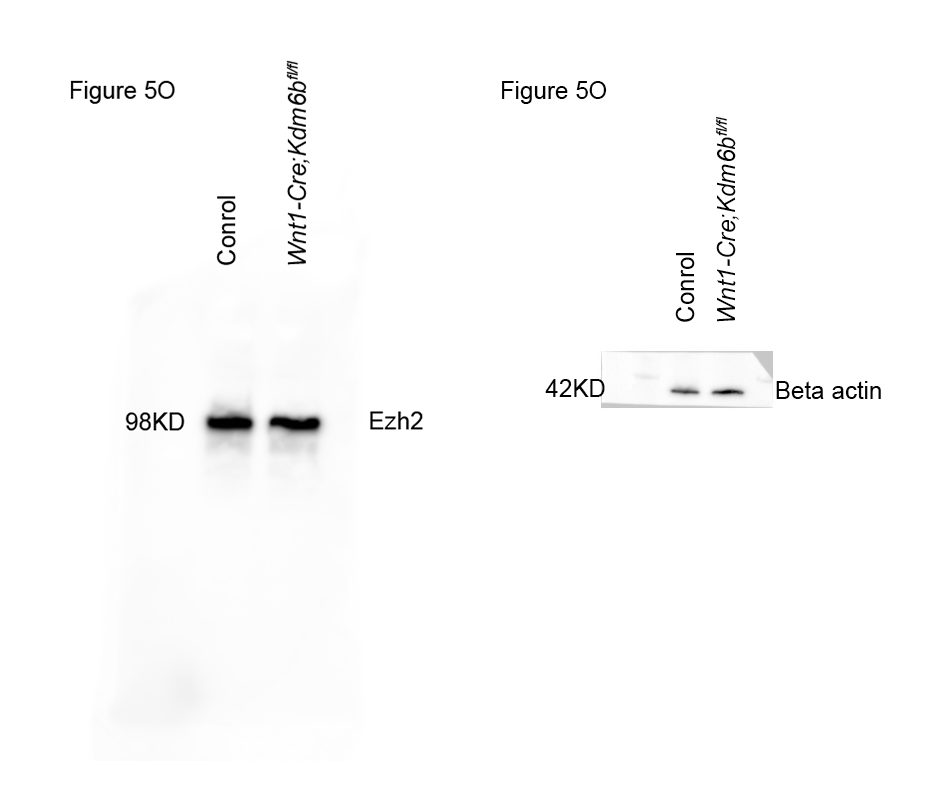

Supplement: Figure 5—source data 3. [file elife-74595-fig5-data3.zip › Figure 5-source data 3 for 5O/Figure 5-source data 10 for Figure 5O.tif]

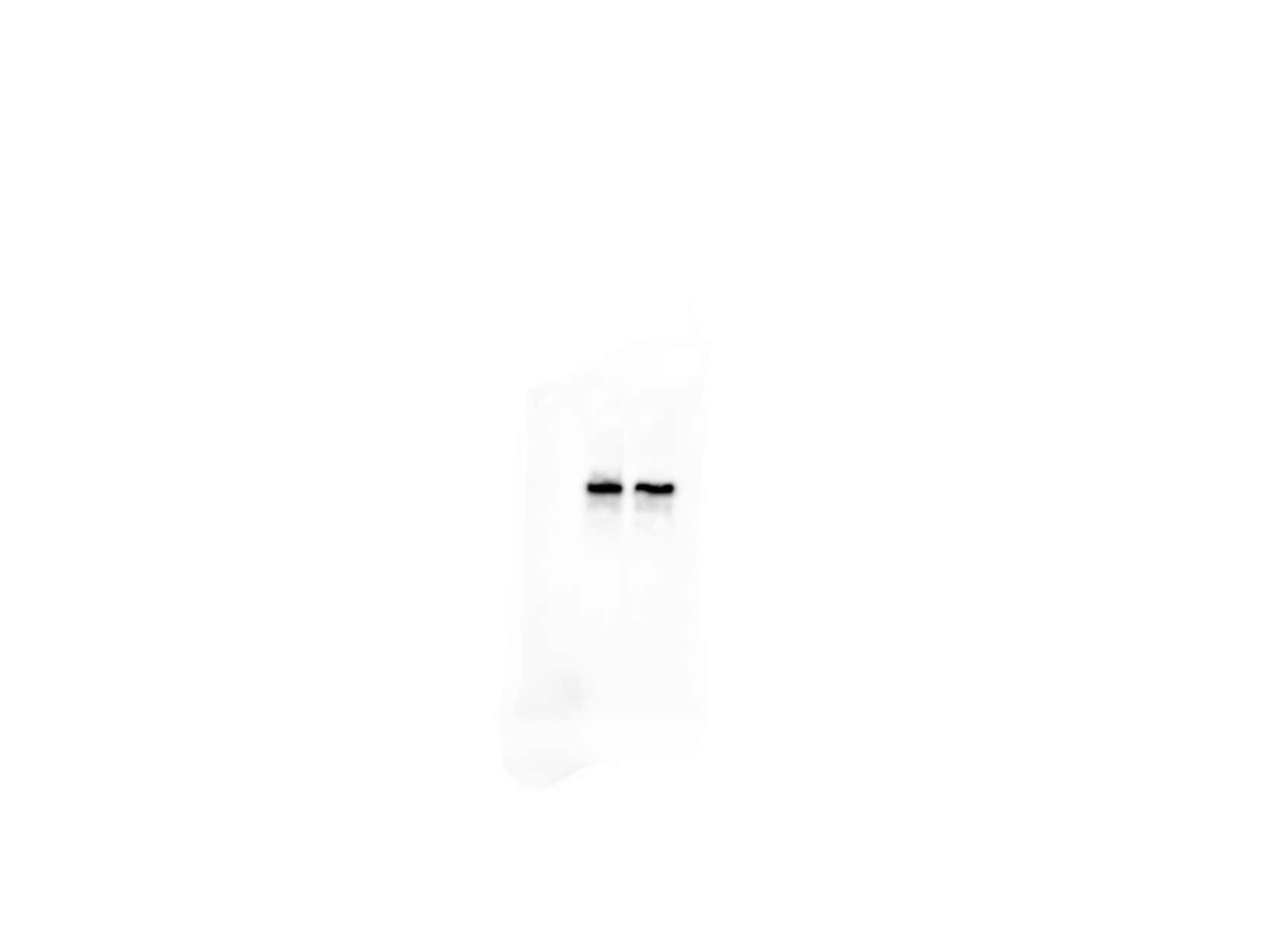

Supplement: Figure 5—source data 3. [file elife-74595-fig5-data3.zip › Figure 5-source data 3 for 5O/Figure 5-source data 8 for Figure 5O.tif]

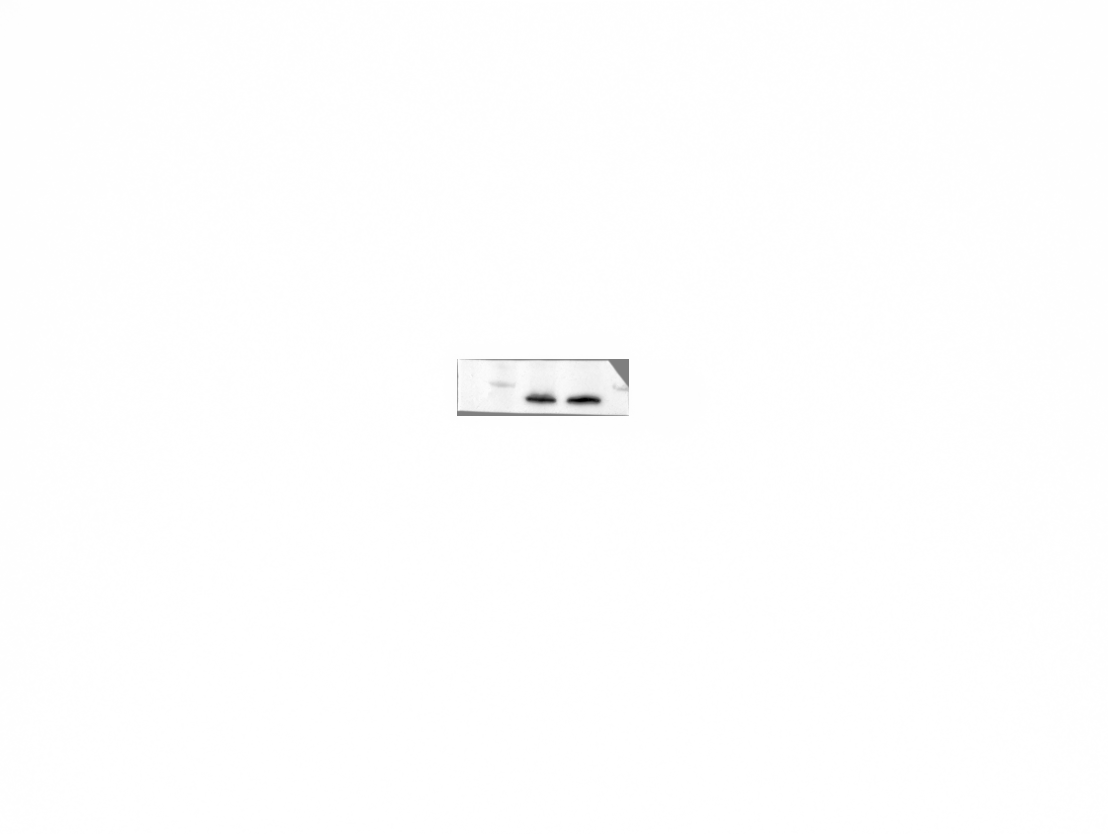

Supplement: Figure 5—source data 3. [file elife-74595-fig5-data3.zip › Figure 5-source data 3 for 5O/Figure 5-source data 9 for Figure 5O.tif]

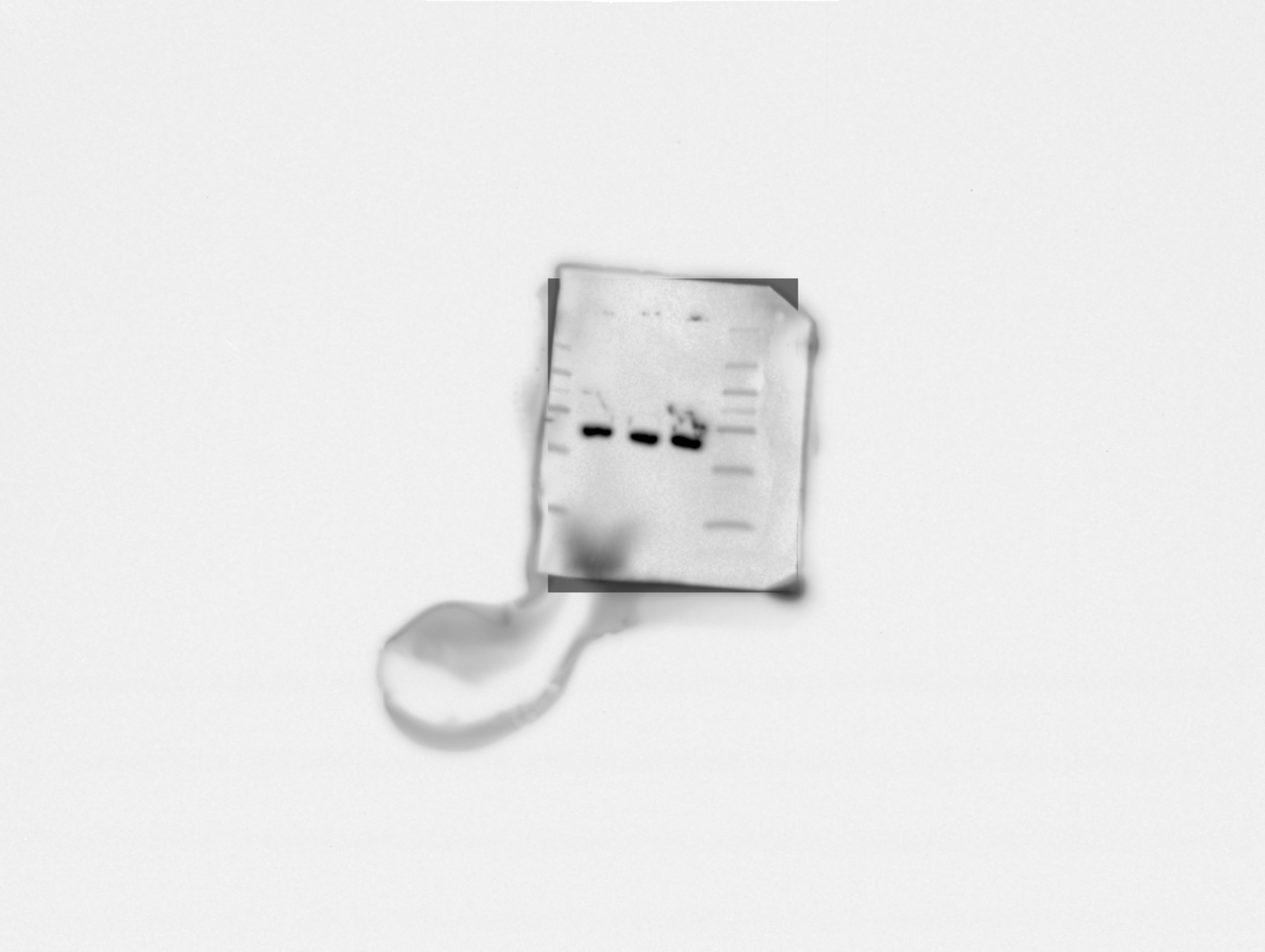

Supplement: Figure 5—source data 4. [file elife-74595-fig5-data4.zip › Figure 5-source data 4 for 5V/Figure 5-source data 11 for Figure 5V.tif]

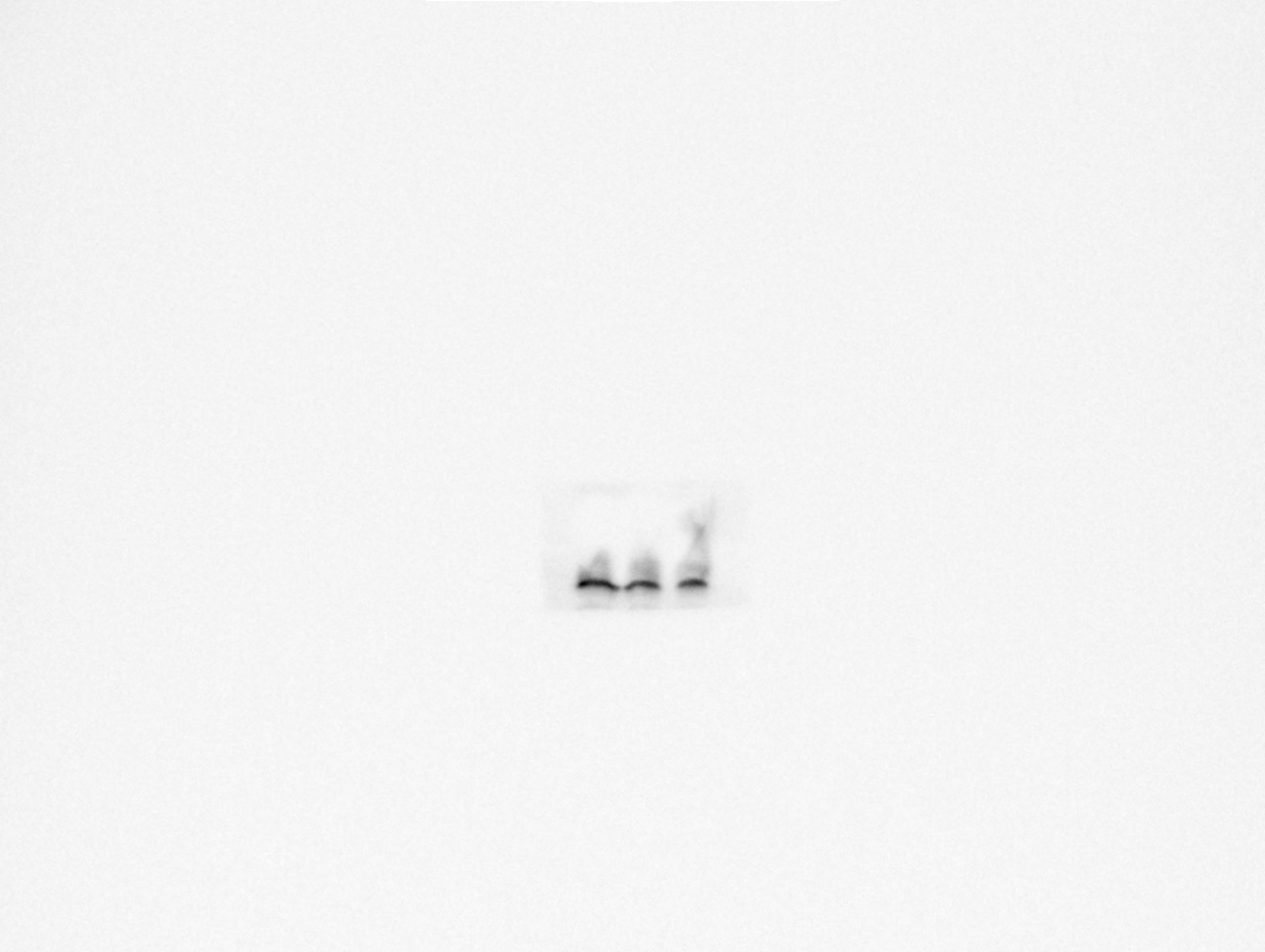

Supplement: Figure 5—source data 4. [file elife-74595-fig5-data4.zip › Figure 5-source data 4 for 5V/Figure 5-source data 12 for Figure 5V.tif]

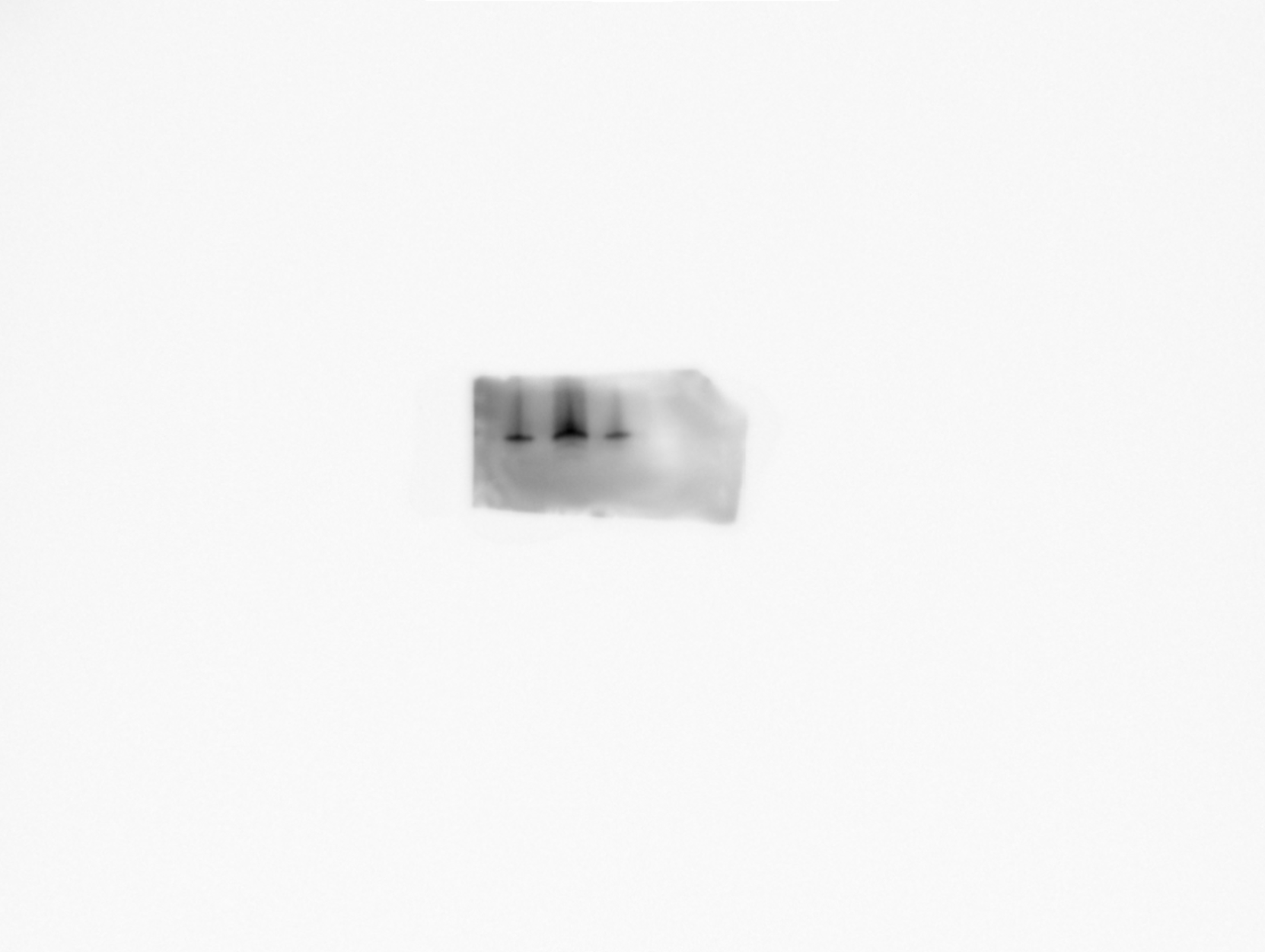

Supplement: Figure 5—source data 4. [file elife-74595-fig5-data4.zip › Figure 5-source data 4 for 5V/Figure 5-source data 13 for Figure 5V.tif]

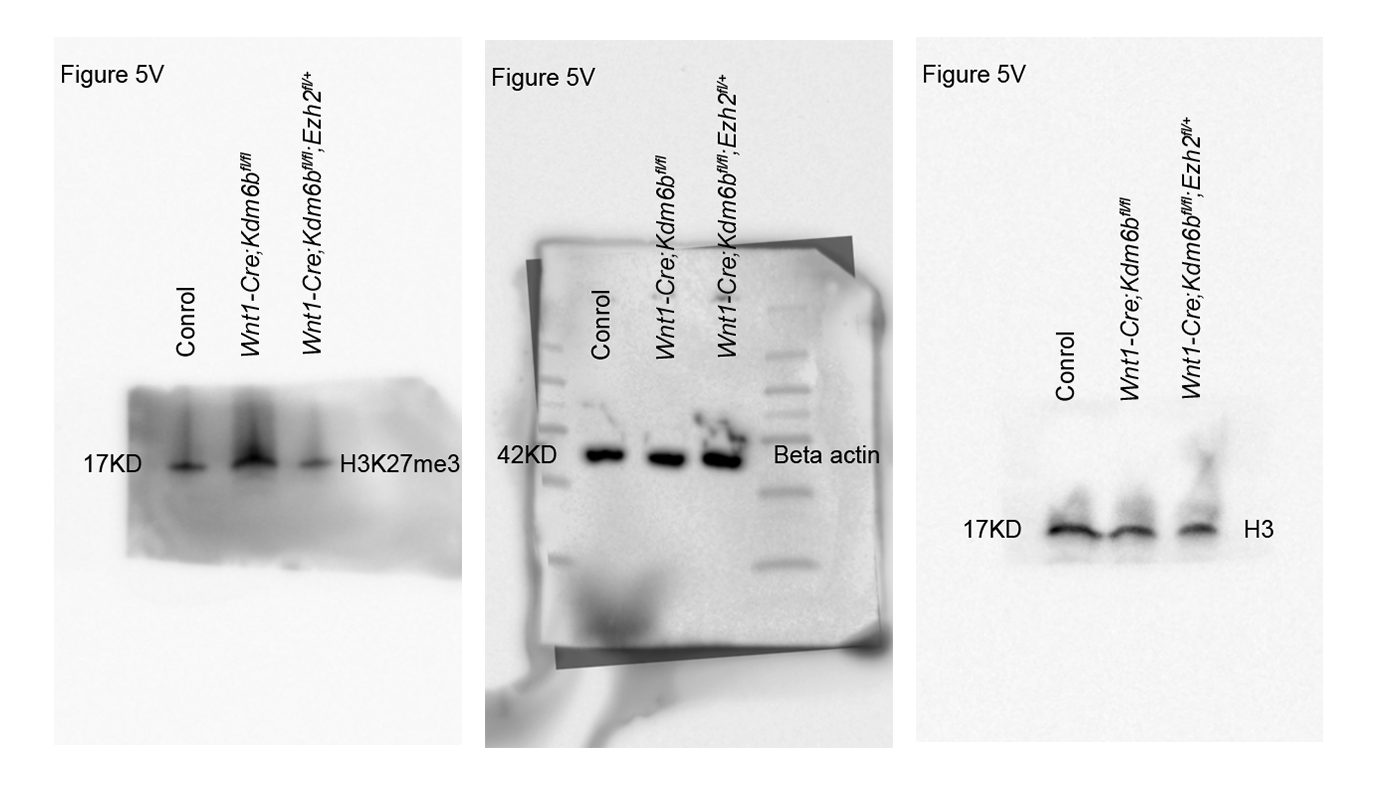

Supplement: Figure 5—source data 4. [file elife-74595-fig5-data4.zip › Figure 5-source data 4 for 5V/Figure 5-source data 14 for Figure 5V.tif]

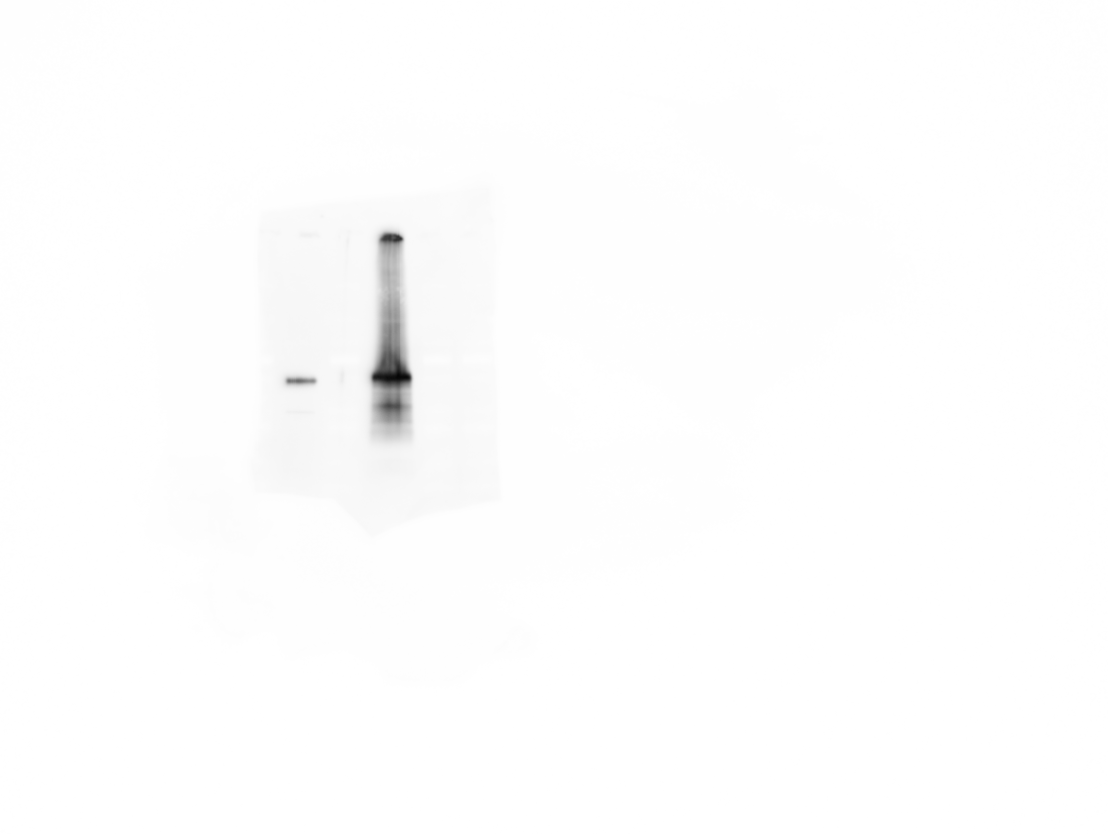

Supplement: Figure 8—source data 11. [file elife-74595-fig8-data11.zip › Figure 8-source data 11 for 8T/Figure 8-source data 11 for Figure 8T .tif]

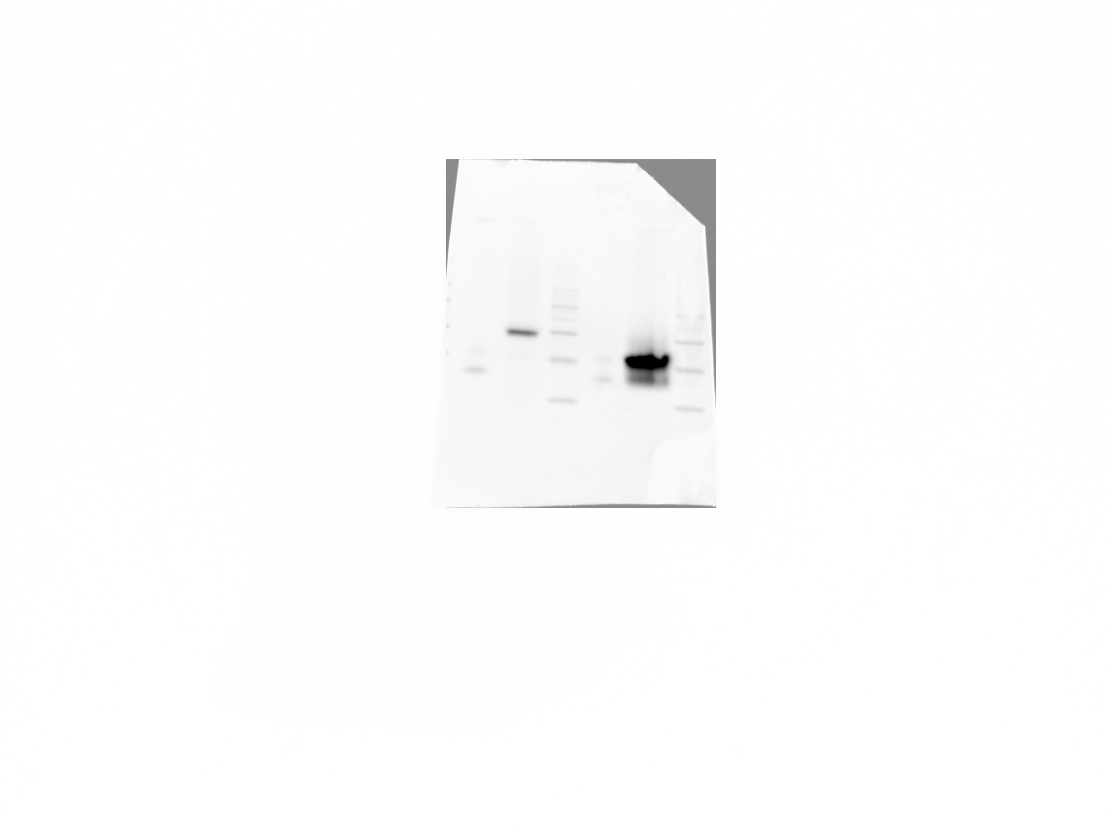

Supplement: Figure 8—source data 11. [file elife-74595-fig8-data11.zip › Figure 8-source data 11 for 8T/Figure 8-source data 12 for Figure 8T.tif]

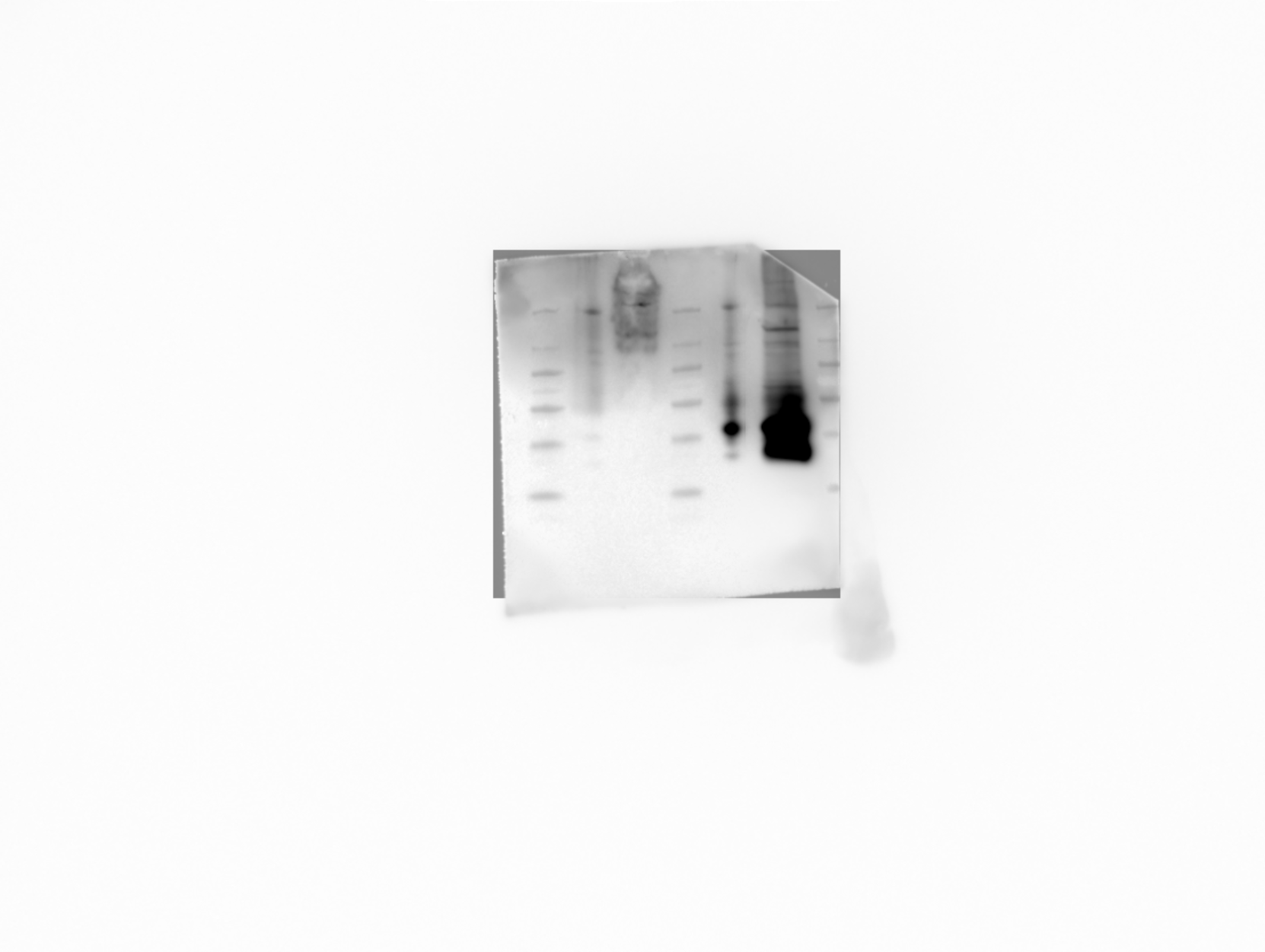

Supplement: Figure 8—source data 11. [file elife-74595-fig8-data11.zip › Figure 8-source data 11 for 8T/Figure 8-source data 13 for Figure 8T.tif]

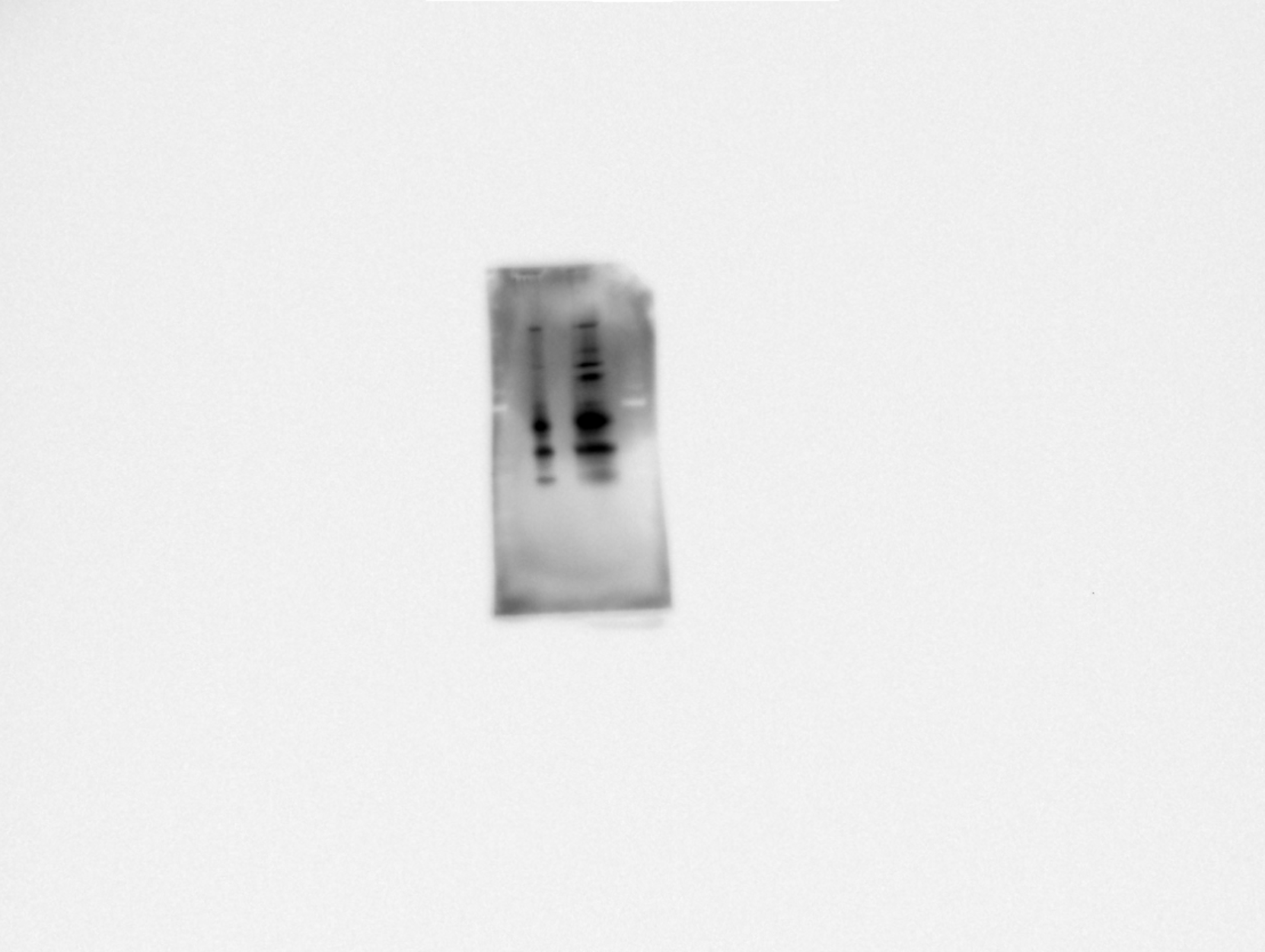

Supplement: Figure 8—source data 11. [file elife-74595-fig8-data11.zip › Figure 8-source data 11 for 8T/Figure 8-source data 14 for Figure 8T.tif]

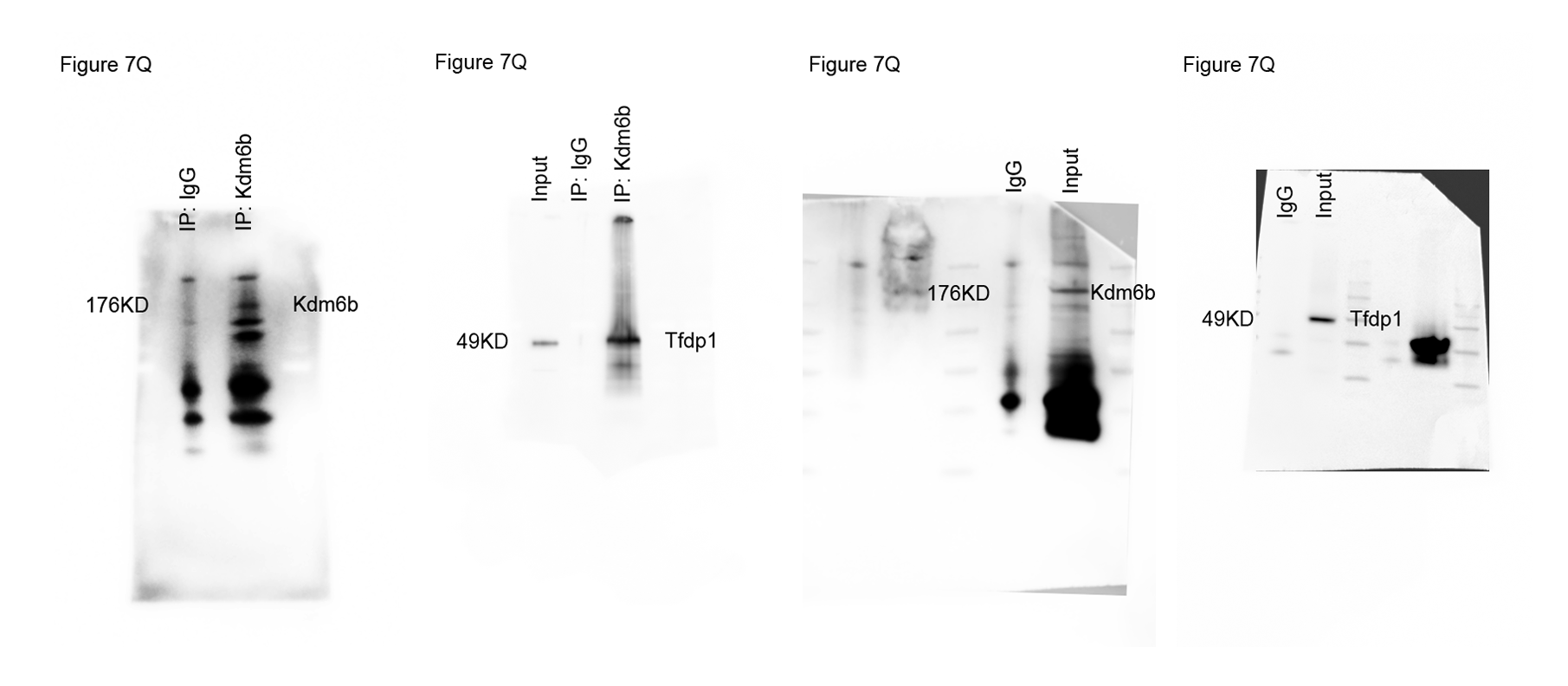

Supplement: Figure 8—source data 11. [file elife-74595-fig8-data11.zip › Figure 8-source data 11 for 8T/Figure 8-source data 15 for Figure 8T.tif]
