# Supplementary material for: KDM6B interacts with TFDP1 to activate P53 signaling in regulating mouse palatogenesis
Source: eLife. 2022 Feb 25;11:e74595. doi: 10.7554/eLife.74595 (PMC9007587; doi:10.7554/eLife.74595)
Supplement: Supplementary file 1. [file elife-74595-supp1.docx]

**Supplementary File 1**

| **Antibodies** | **Vendor** | **Cat No.** | **Dilution** |
| --- | --- | --- | --- |
| myosin heavy chain (MHC) | DSHB | P13538 | 1:10 |
| Histone H3 tri methyl K27 (H3K27me3) | Cell signaling | 9733s | 1:200 |
| Phospho-Histone H2A.X (Ser139) | Cell signaling | 9718s | 1:200 |
| DP1 | Abcam | ab124678 | 1:100 |
| EZH2 | Cell Signaling | 5246s | 1:200 |
| RUNX2 | Cell Signaling | 12556s | 1:200 |
| SP7 | Abcam | ab22552 | 1:200 |
| Lamin B1 | Cell Signaling | 17416s | 1:100 |
| Alexa Fluor 568 | Invitrogen | A-11011 | 1:200 |
| Alexa Fluor 488 | Invitrogen | A-32931 | 1:200 |
