# Supplementary material for: KDM6B interacts with TFDP1 to activate P53 signaling in regulating mouse palatogenesis
Source: eLife. 2022 Feb 25;11:e74595. doi: 10.7554/eLife.74595 (PMC9007587; doi:10.7554/eLife.74595)
Supplement: Supplementary file 2. [file elife-74595-supp2.docx]

**Supplementary File 2**

| **Probes** | **Vendor** | **Cat No.** |
| --- | --- | --- |
| *Kdm6a* | Advanced Cell Diagnostics | 456961 |
| *Kdm6b* | Advanced Cell Diagnostics | 477971 |
| *Kdm6b-01* | Advanced Cell Diagnostics | 501231 |
| *Uty* | Advanced Cell Diagnostics | 451741 |
| *Trp53* | Advanced Cell Diagnostics | 402331 |
| *Trp53-C2* | Advanced Cell Diagnostics | 402331-C2 |
| *Ezh1* | Advanced Cell Diagnostics | 895231 |
| *Wrap53* | Advanced Cell Diagnostics | 1143201-C1 |
