# Supplementary material for: KDM6B interacts with TFDP1 to activate P53 signaling in regulating mouse palatogenesis
Source: eLife. 2022 Feb 25;11:e74595. doi: 10.7554/eLife.74595 (PMC9007587; doi:10.7554/eLife.74595)
Supplement: Supplementary file 3. [file elife-74595-supp3.docx]

**Supplementary File 3**

| **Genes** |  | **Primer sequence** |
| --- | --- | --- |
| *Trp53* ChIP-qPCR | Forward | 5'-AGGTCAGGAGGGAGGCTATC-3' |
|  | Reverse | 5'-GCTTTGGACACTCGTTCCCT-3' |
| *Trp53* RT-qPCR | Forward | 5'-GTGTGGTGCAGATCGCAGT-3' |
|  | Reverse | 5'-ATCATGCCTTCGGACTTGATG-3' |
| *Gapdh* RT-qPCR | Forward | 5'-TGGATTTGGACGCATTGGTC-3' |
|  | Reverse | 5'-TTTGCACTGGTACGTGTTGAT-3' |
| *Tfdp1* RT-qPCR | Forward | 5'-TTGAAGCCAACGGAGAACTAAAG-3' |
|  | Reverse | 5'-TGGACTGTCCGAAGGTTTTTG-3' |
| *Runx2* RT-qPCR | Forward | 5'-AGAGTCAGATTACAGATCCCAGG-3' |
|  | Reverse | 5'-TGGCTCTTCTTACTGAGAGAGG-3' |
| *Sp7* RT-qPCR | Forward | 5'-AAGTCTCAAGGTTATAGGGACGG-3' |
|  | Reverse | 5'-CCATGCTTGTCTGGGTATAGTGT-3' |
| *Kdm6a* RT-qPCR | Forward | 5'-CGGGCGGACAAAAGAAGAAC-3' |
|  | Reverse | 5'- CATAGACTTGCATCAGATCCTCC-3' |
| *Kdm6b* RT-qPCR | Forward | 5'-TGAAGAACGTCAAGTCCATTGTG-3' |
|  | Reverse | 5'-TCCCGCTGTACCTGACAGT-3' |
