# Supplementary material for: KDM6B interacts with TFDP1 to activate P53 signaling in regulating mouse palatogenesis
Source: eLife. 2022 Feb 25;11:e74595. doi: 10.7554/eLife.74595 (PMC9007587; doi:10.7554/eLife.74595)
Supplement: Supplementary file 4. [file elife-74595-supp4.docx]

**Supplementary File 4**

| **Antibodies** | **Vendor** | **Cat No.** | **Dilution** |
| --- | --- | --- | --- |
| Histone H3 tri methyl K27 (H3K27me3) | Cell signaling | 9733s | 1:1000 |
| DP1 | Abcam | ab124678 | 1:1000 |
| EZH1 | Abcam | ab189833 | 1:1000 |
| EZH2 | Cell Signaling | 5246s | 1:2000 |
| KDM6A | Abcam | ab36938 | 1:1000 |
| KDM6B (C-term) | Abcepta | AP1022b | 1:1000 |
| KDM6B (N-term) | Abcepta | AP1022a | 1:1000 |
| P53 | Santa Cruz | sc-126 | 1:1000 |
| Histone H3 | Cell signaling | 4499s | 1:1000 |
| Beta Actin | Abcam | ab20272 | 1:2000 |
| Rabbit IgG HRP-conjugated antibody | R&D System | HAF008 | 1:2000 |
| Mouse IgG HRP-conjugated antibody | R&D System | HAF007 | 1:2000 |
| HRP, Mouse Anti-Rabbit IgG LCS | IPKine™ | A25022 | 1:2000 |
