# Supplementary material for: KDM6B interacts with TFDP1 to activate P53 signaling in regulating mouse palatogenesis
Source: eLife. 2022 Feb 25;11:e74595. doi: 10.7554/eLife.74595 (PMC9007587; doi:10.7554/eLife.74595)
Supplement: Supplementary file 5. [file elife-74595-supp5.docx]

**Supplementary File 5**

| **Product name** | **Cat No.** | **Target sequence** |
| --- | --- | --- |
| Mm_Trp53_5 | SI04394257 | CTGGGACAGCCAAGTCTGTTA |
| Mm_Trp53_4 | SI01456532 | TGGAGAGTATTTCACCCTCAA |
| Mm_Trp53_3 | SI01456525 | ACCGCCGTACAGAAGAAGAAA |
| Mm_Trp53_1 | SI01456511 | CCGGGTGGAAGGAAATTTGTA |
| Mm_Tfdp1_7 | SI02710834 | CAGAATCTTAGTCCTGGGAAA |
| Mm_Tfdp1_6 | SI02688840 | AAAGGTCTTTATAGACCAGAA |
| Mm_Tfdp1_5 | SI02668918 | CACACCAGTGACAATGACAAA |
| Mm_Tfdp1_1 | SI00183995 | AACGACGAGGAGGATTGATTA |
| AllStars Negative Control siRNA | 1027280 |  |
