# Supplementary material for: KDM6B interacts with TFDP1 to activate P53 signaling in regulating mouse palatogenesis
Source: eLife. 2022 Feb 25;11:e74595. doi: 10.7554/eLife.74595 (PMC9007587; doi:10.7554/eLife.74595)
Supplement: Supplementary file 6. [file elife-74595-supp6.docx]

**Supplementary File 6**

| **Product name** | **Vendor** | **Cat No.** |
| --- | --- | --- |
| pCMV6-AC-GFP, mammalian vector | OriGene | PS100010 |
| pCMV6-Entry, mammalian vector | OriGene | PS100001 |
| *Tfdp1* (NM_009361) Mouse Tagged ORF Clone | OriGene | MG221670 |
| *Kdm6a* (NM_009483) Mouse Tagged ORF Clone | OriGene | MG211981 |
| *Kdm6b* (NM_001017426) Mouse Tagged ORF Clone | OriGene | MR212060 |
| Turbo Fectin Tranfection Reagent | OriGene | TF81001 |
