# Supplementary material for: KDM6B interacts with TFDP1 to activate P53 signaling in regulating mouse palatogenesis
Source: eLife. 2022 Feb 25;11:e74595. doi: 10.7554/eLife.74595 (PMC9007587; doi:10.7554/eLife.74595)
Supplement: Supplementary file 7. [file elife-74595-supp7.docx]

**Supplementary File 7**

| **Pre-alignment qa/qc** | | | | | |
| --- | --- | --- | --- | --- | --- |
| Sample name | Total reads | Read length | Avg. read quality | % N | % GC |
| 6B-WT_S3 | 117089731 | 76 | 33.4671 | 0.046936 | 44.9143 |

| **Post-alignment qa/qc** | | | | | | | | | | |
| --- | --- | --- | --- | --- | --- | --- | --- | --- | --- | --- |
| Sample name | Total reads | Total alignments | Aligned | Total unaligned | Unaligned | Total unique singleton | Unique singleton | Total unique paired |  |  |
| 6B-WT_S3 | 1E+08 | 2E+08 | 97.265 | 3E+06 | 2.7351 | 1951709 | 1.66685 | 1.1E+08 |  |  |
| Unique paired | Total non-unique paired | Non-unique paired | Total non-unique singleton | Non-unique singleton | Coverage | Avg. coverage depth | Avg. length | Avg. quality | %GC |  |
| 94.96082 | 701598 | 0.599 | 44522 | 0.038 | 80.327 | 7.09937 | 75.8895 | 33.6187 | 45.1441 |  |
